# Solvent‐Rich Pre‐Coagulation Bath for Tunable Liquid‐State Fusion Enables Robust Two‐Step Polymer Embedded Printing

**DOI:** 10.1002/advs.202508335

**Published:** 2025-07-28

**Authors:** Kaidong Song, Qian Wu, Ashley M. Compaan, Junting Shen, Chuanshen Zhou, Mingwei Chen, Marc Sole‐Gras, John‐Thomas T. Robinson, Bing Ren, Huayong Yang, Yong Huang, Jun Yin

**Affiliations:** ^1^ Department of Mechanical and Aerospace Engineering University of Florida Gainesville FL 32611 USA; ^2^ The State Key Laboratory of Fluid Power and Mechatronic Systems School of Mechanical Engineering Zhejiang University Hangzhou 310028 China; ^3^ Department of Materials Science and Engineering University of Florida Gainesville FL 32611 USA

**Keywords:** embedded 3D printing, engineering polymers, immersion phase separation, liquid‐state fusion, solvent‐rich support bath

## Abstract

Effective interlayer fusion is a critical step enabling a 3D printing process. For engineering polymer printing, interlayer fusion is usually either heat‐enabled or binder‐based, which may introduce thermal residual stress, warpage, and undesirable impurities. To overcome such challenges, a two‐step immersion phase separation (IPS)‐based room‐temperature polymer fusion and solidification approach for self‐supported engineering polymer printing, termed IPS‐embedded 3D printing (IPS‐E3DP) is introduced. IPS‐E3DP is implemented by first depositing polymer inks in a solvent‐rich yield‐stress pre‐coagulation support bath to have an intermediate green part and then immersing the intermediate green part in a non‐solvent coagulation bath for complete solidification. During the first critical step, dissolved polymer ink holds its deposited shape and fuses with a previously deposited polymeric feature in the pre‐coagulation support bath. Specifically, it is the high‐concentration solvent bath (usually 80% or higher) that enables tunable liquid‐state fusion of polymers when the deposited polymer solidifies, and it is the yield‐stress support bath that prevents the deposited polymer ink from spreading and deformation during fusion. IPS‐E3DP enables the self‐supported room‐temperature high‐fidelity printing of a wide range of engineering polymers, blends, and composites with superior geometric complexity. The process can be tailored as a polymer binder‐based printing approach for versatile material printing.

## Graphical abstract

Additive manufacturing enables complex structures, but polymer printing often suffers from poor interlayer fusion and defects induced by heat or binders. A two‐step, room‐temperature strategy based on immersion phase separation (IPS‐E3DP) achieves precise liquid‐state fusion with high structural fidelity, enhanced mechanical performance, broad material compatibility, and scalable, energy‐efficient processing.

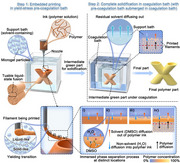

## 1 Introduction

Additive manufacturing, commonly referred to as 3D printing, has provided an advanced approach to creating complex structures from polymers, ceramics, and metals.^[^ ^1^, ^2^, ^3^ ^]^ Generally, a final product is printed in a layer‐by‐layer fashion, necessitating effective fusion mechanisms to create seamless and integral parts.^[^ ^4^, ^5^, ^6^ ^]^ The fusion process can be primarily categorized by the transition of the state of layers involved: liquid‐liquid fusion, solid‐solid fusion, and solid‐liquid fusion. Engineering polymer printing usually adopts a liquid‐liquid fusion process, represented by fused filament fabrication using thermal means to convert it from a solid to a liquid state for thermal bonding. Alternatively, engineering polymers can be printed using a solid‐solid fusion process such as solid‐state sintering, which relies on the heat‐induced diffusion of polymer particles at temperatures below their melting point. Moreover, engineering polymers can be printed through a liquid‐solid fusion process via binder‐assisted chemical adhesion. For the first two fusion processes, engineering polymers need to be heated, which typically comes with thermal residual stress and induced warpage in addition to being energy demanding;^[^ ^7^, ^8^ ^]^ for the third process, a binder is needed, which raises the concern of undesirable impurities.^[^ ^9^, ^10^ ^]^ While stereolithography (SLA) enables high‐resolution, support‐free printing of complex polymer structures, it is fundamentally limited to photocurable resins, restricting its material versatility and suitability for engineering‐grade polymers.^[^ ^11^, ^12^ ^]^ Thus, there is a need for an alternative fusion process for room‐temperature printing of engineering polymers that avoids thermal warpage and binder‐related impurities.

**Figure 1:**
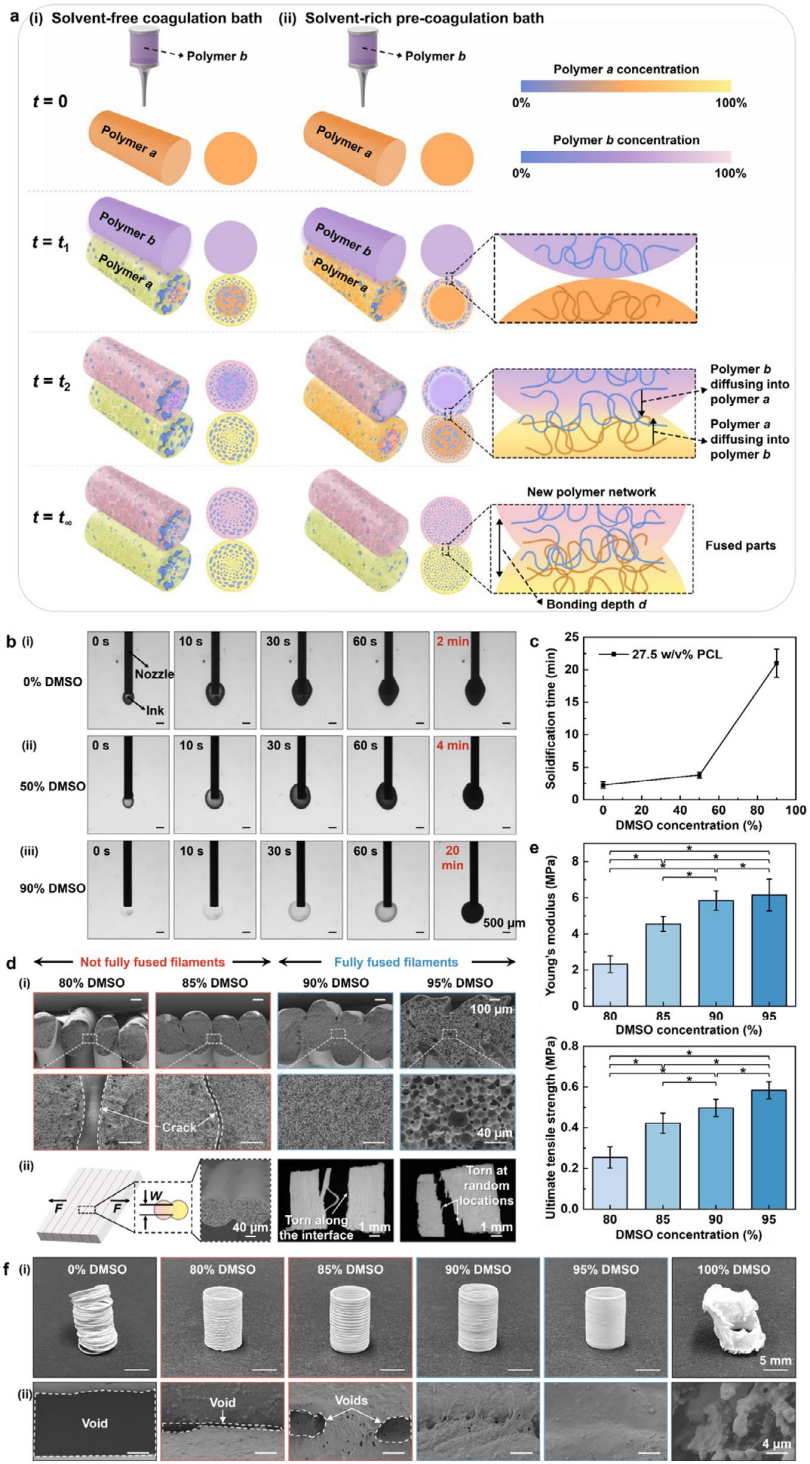
Liquid‐state polymer fusion studies. a) Schematic of polymer‐polymer diffusion process: (i) solvent‐free coagulation bath versus (ii) solvent‐rich pre‐coagulation bath. b) Pictures and c) solidification time of polymer‐based ink in coagulation/pre‐coagulation baths with different DMSO concentrations. d) (i) Cross‐sectional morphology of inter‐filament fusion under different DMSO concentrations and (ii) tensile testing results for inter‐filament fusion evaluation. e) Mechanical properties of samples printed with different DMSO concentrations. f) Tubular‐shaped structures printed with different DMSO concentrations, showing (i) optical images and (ii) SEM‐surface views.

For effective fusion during room‐temperature polymer printing, the transition from solid to liquid before printing and from liquid to solid during printing is expected at room temperature. This could be accomplished by utilizing the physics that solid polymeric structures can be effectively fabricated from polymer solutions using various phase separation methods based on the chemical potential gradients provoked by changes in the composition of a homogeneous polymeric system.^[^ ^13^, ^14^ ^]^ Particularly, when a homogenous polymer solution meets a non‐solvent‐based coagulant, the polymer experiences a reduction in solubility as the solvent is gradually replaced by the non‐solvent, causing the polymer to demix into regions with high and low polymer concentrations,^[^ ^15^, ^16^ ^]^ which represents a typical immersion phase separation (IPS) mechanism. The polymer transition process from a dissolved state in the solvent to a solid state as it precipitates out of the solution may be utilized for tunable fusion between a solid polymer structure and a polymer solution. As such, the IPS mechanism can be tailored to print polymeric structures as a new fusion mechanism under which the dissolved polymer contacts a previously deposited polymeric substrate and fuses over the polymeric substrate (**Figure** **1**a). This leads to an efficient fusion joining mechanism for polymer product manufacturing, which has been utilized for polymer printing applications as initially proposed by us^[^ ^17^, ^18^ ^]^ and the Hashimoto group^[^ ^19^ ^]^ independently around 2019.^[^ ^18^, ^19^, ^20^, ^21^, ^22^ ^]^ However, a key challenge remains—balancing the fluidity of deposited polymer ink to facilitate fusion while preventing undesired spreading. This limitation has hindered the broader adoption of IPS‐based printing, restricting prior demonstrations to discrete filamentous or simple structures,^[^ ^20^, ^21^, ^22^ ^]^ without achieving high‐fidelity, continuous 3D complex geometries with smooth surfaces. Addressing this challenge is crucial to unlocking the full potential and adoption of IPS‐based polymer printing for advanced manufacturing applications.

To address the aforementioned challenge, we propose a two‐step printing approach by first depositing polymer inks in a solvent‐rich yield‐stress pre‐coagulation support bath to have an intermediate part and then immersing the pre‐coagulation bath and intermediate green part in a non‐solvent coagulation bath for complete solidification instead of depositing into a non‐solvent coagulation bath directly.^[^ ^19^, ^20^, ^21^, ^22^ ^]^ Since the fusion and solidification rate of the deposited polymer ink is intricately linked to the availability of solvent and non‐solvent,^[^ ^16^ ^]^ our approach has particularly addressed the filament fusion challenge by incorporating a high‐concentration solvent (such as 80% or higher) in the pre‐coagulation support bath as the first printing step. This critical step enables the control and balance of polymer liquid‐state fusion (a state wherein the polymers are partially solidified but retain sufficient fluidity and molecular mobility to facilitate effective fusion) and solidification, and the solvent‐containing pre‐coagulation support bath (including a mixture of solvent and non‐solvent) warrants a tunable liquid‐state fusion but limited phase‐separation process for constructing intricate structures. Moreover, the use of a yield‐stress bath^[^ ^23^, ^24^, ^25^, ^26^, ^27^ ^]^ effectively confines polymer inks in situ, maintaining their deposited shape throughout the IPS process (a self‐supported printing property) and ensuring seamless liquid‐state fusion of polymeric bodies without undesirable spreading and diffusion of ink. This approach provides a solid framework for achieving detailed and reliable polymer fusion in additive manufacturing, enabling robust self‐supported polymer embedded printing.

## 2 Results and Discussion

### 2.1 Liquid‐State Polymer Fusion in Solvent‐Rich Pre‐Coagulation Bath

During IPS‐enabled polymer fabrication, sequential deposition into a coagulation bath induces complex phase separation governed by the ternary Cahn–Hilliard equation.^[^ ^28^, ^29^ ^]^ As a result of the phase separation process, the solidification degree of each polymer solution heavily depends on the phase separation rate. When the polymer in the polymer solution *a*, which may be solidified or solidifying, comes in contact with a subsequently deposited polymer solution *b*, the initial composition of the coagulation bath significantly influences the solidification and subsequent interaction of the polymer solutions of *a* and *b* as determined by the interdiffusion between two polymers (Figure 1a). In a solvent‐free coagulation bath (strictly speaking, solvent‐lean coagulation bath since there may be some low‐quantity solvent extracted from polymer solutions), polymer *a* undergoes instantaneous demixing upon immersion, solidifying instantaneously before being in contact with polymer *b* (Figure 1a (i), 0 < *t* < *t_1_*). This results in the formation of a dense, possibly less permeable layer in polymer *a*, impeding the diffusion of polymer *b* into polymer *a* and leading to weak interfacial bonding or even complete separation between two adjacent polymers. Conversely, a solvent‐rich bath as a pre‐coagulation one results in a slower phase separation process of polymer *a*, which gives the polymer *b* solution sufficient time to diffuse into semi‐solid or still‐fluid polymer *a* and vice versa, enhancing interfacial bonding and increasing the likelihood of forming a cohesive structure (Figure 1a (ii)). The diffusion of polymer chains across the interface between the polymer solutions of *a* and *b* is driven by the concentration gradients and polymer chain mobility, following Fick's law as:

(1) $$\frac{\partial C_{a}}{\partial t}=D_{ab}\;\frac{\partial^{2}C_{a}}{\partial z^{2}}$$

where *C_a_* is the concentration of polymer *a* at position *z* and time *t*, and *D_ab_* represents the diffusion coefficient for polymer chains from polymer *a* to polymer *b* at time *t*. This diffusion coefficient, or the diffusion rate and extent, depends on the mobility of polymer chains, which is significantly influenced by the solidification degree and the viscosity of polymer solutions of *a* and *b*. Some representative simulation results can be seen in Note S1 (Supporting Information). The fusion of polymers *a* and *b* initiates with the surface contact (*t* = *t*_1_), leading to intermolecular diffusion that causes chain segments from each polymer to intertwine at the interface. This interaction results in entanglements and possibly chemical bonds, and its efficiency is affected by polymer miscibility and molecular compatibility. The resulting interfacial bonding strength is closely linked to the bonding depth (*d* in Figure 1a (ii), *t* = *t_∞_*), determined by the time‐dependent diffusion coefficient *D_ab_*. Simulation results in Figure S3 (Supporting Information) indicate that an increase in the diffusion coefficient directly leads to a greater bonding depth over a given period, underscoring the significance of controlling the diffusion coefficient or solvent concentration to enhance the liquid‐state polymer fusion efficiency during IPS.

To appreciate the addition of solvent in the pre‐coagulation bath, the solvent concentration was experimentally tuned to characterize its influence on the liquid‐state fusion and solidification process of polymer solutions. For demonstration, dimethyl sulfoxide (DMSO) was selected as a solvent for its recyclability, low toxicity, and effective solubility with common engineering polymers (Note S2, Supporting Information). Polycaprolactone (PCL) was chosen as a model polymer, and the polymer solidification rate was characterized using optical imaging, and acrylonitrile butadiene styrene (ABS) was selected to investigate the effect of DMSO concentration on the polymer interdiffusion process in the pre‐coagulation bath. The pre‐coagulation bath was a DMSO and water (as non‐solvent for polymers)‐supplemented poly(acrylic acid) (Carbopol)‐based yield‐stress bath. Figure 1b visualizes the PCL solidification rate as a function of DMSO concentration when the PCL was extrusion dispensed into a DMSO‐included pre‐coagulation bath (0%, 50%, and 90% DMSO). The PCL ink changes from transparent to opaque as the solidification process goes on, and the required solidification time changes from 2.28 ± 0.49 min to 3.79 ± 0.46 min to 21.00 ± 2.16 min as the DMSO concentration increases (Figure 1c), indicating a reduced solidification rate. A higher solvent concentration in the bath slows the solvent and non‐solvent exchange process and, consequently, the solidification rate of polymers. Notably, this slower solidification rate is a key feature of our method, enabling controlled liquid‐state fusion and enhanced interlayer bonding without compromising printing speed, and can be tuned for different material systems. The biocompatibility of printed structures is further evidenced by the successful bacterial colonization and biomineralization in Figure 6, indicating the successful removal of DMSO in printed structures. Although solvent recycling was not implemented in this study, established techniques such as distillation and membrane separation offer feasible routes for future process sustainability.

The interdiffusion process‐induced fusion of two adjacent filaments was further investigated during deposition an ABS ink. Figure 1d depicts the degree of fusion under different DMSO concentrations. When 80% or 85% DMSO is used, there are visible interfaces between the filaments; when 90% or 95% DMSO is used, the filaments are fused well with each other. It can be seen that the inter‐filament fusion is significantly enhanced by the higher concentration DMSO baths, which is attributed to the reduced DMSO concentration gradient between the bath and ink. As a result, the solvent and non‐solvent exchange process is slowed down, enhancing filament fusion with the previously deposited polymer. As seen in Figure 1d (i), the fusion mechanism is a liquid‐liquid one since the filament interface is indiscernible (fully fused filaments); otherwise, the interface is visible under the solid‐liquid fusion mechanism (not fully fused filaments). In addition, Figure 1d (ii) shows the schematic of the tensile test of fused filaments with a force (*F*) and the resulting fractured interface: torn along the filament interface for not fully fused filaments and torn at random locations across the fused filaments for fully fused filaments. The latter indicates a good fusion quality and larger interlayer fusion width (*W*) among the filaments.

It is further noted that an appropriate solvent concentration may induce uniform pores in polymers during the phase separation process (Figure 1d; Figure S1b,c, Supporting Information, 90% DMSO for ABS or ABS/thermoplastic polyurethane (TPU) ink) by ensuring a controlled, gradual solvent/non‐solvent exchange for spatially uniform phase separation and further the formation of evenly distributed polymer‐rich and polymer‐poor phases. Such a controlled process is essential for achieving pore monodispersity throughout the polymer, preventing pore irregularities caused by either too rapid or insufficient phase separation. Therefore, determining a proper solvent concentration is critical to producing polymers with well‐fused and uniformly porous morphology. This feature not only facilitates the creation of intricate macro‐scale designs but also allows precise control over micro‐scale fusion and porosity within structures, as effectively demonstrated in a schematic representation (Figure S1, Supporting Information).

Furthermore, ABS planar sheet structures were first deposited in a DMSO‐rich yield‐stress pre‐coagulation support bath to form a structure and further solidified in a water‐only coagulation bath. Their tensile mechanical properties, shown in Figure 1e, correlated with DMSO concentration: a higher DMSO concentration results in higher (stiffer or stronger) Young's modulus and ultimate tensile strength. Such variations can be linked to the value of the diffusion coefficient *D_ab_*, as described in Equation (1), as well as the bonding depth (*d*), illustrated in Figure 1a (ii). In solvent‐rich pre‐coagulation support baths, the increased diffusion coefficient facilitates the intermolecular diffusion and intertwining of polymer chains, leading to improved inter‐filament fusion. The bonding depth, which reflects the extent of polymer chain interaction at the interface, also plays a crucial role in influencing the mechanical properties. Deeper bonding, indicating more intensive chain entanglements and a chance of robust bonds, affects the mechanical properties of fused polymers. Furthermore, the pore monodispersity, as observed in higher solvent concentration baths (Figure 1d), ensures consistent mechanical properties across polymeric structures, which may augment their structural integrity and durability. This suggests that using a solvent‐rich pre‐coagulation support bath can enhance the mechanical properties of polymers due to improved inter‐filament fusion.

The solvent concentration also clearly influences the shape and integrity of polymer structures, as seen in Figure 1f, in particular, those scanning electron microscopy (SEM) images of the resultant surfaces under different DMSO concentrations. The structures formed in the pre‐coagulation support baths with higher solvent concentrations (e.g., 95% DMSO) show greater integrity and better shape fidelity, reflecting the similar fusion quality observations as seen in Figure 1d (i). Further analysis of the shape fidelity is provided in Figures S4–S7 (Supporting Information). It is noted that if the yield‐stress pre‐coagulation support bath has 100% DMSO and the phase‐separation process happens during the post‐deposition solidification process, the resulting structure may be distorted, as seen in Figure 1f, and is not recommended. That is, minimal phase separation and solidification are expected during the structure‐forming process.

### 2.2 Two‐Step Immersion Phase Separation Embedded 3D Printing

**Figure 2:**
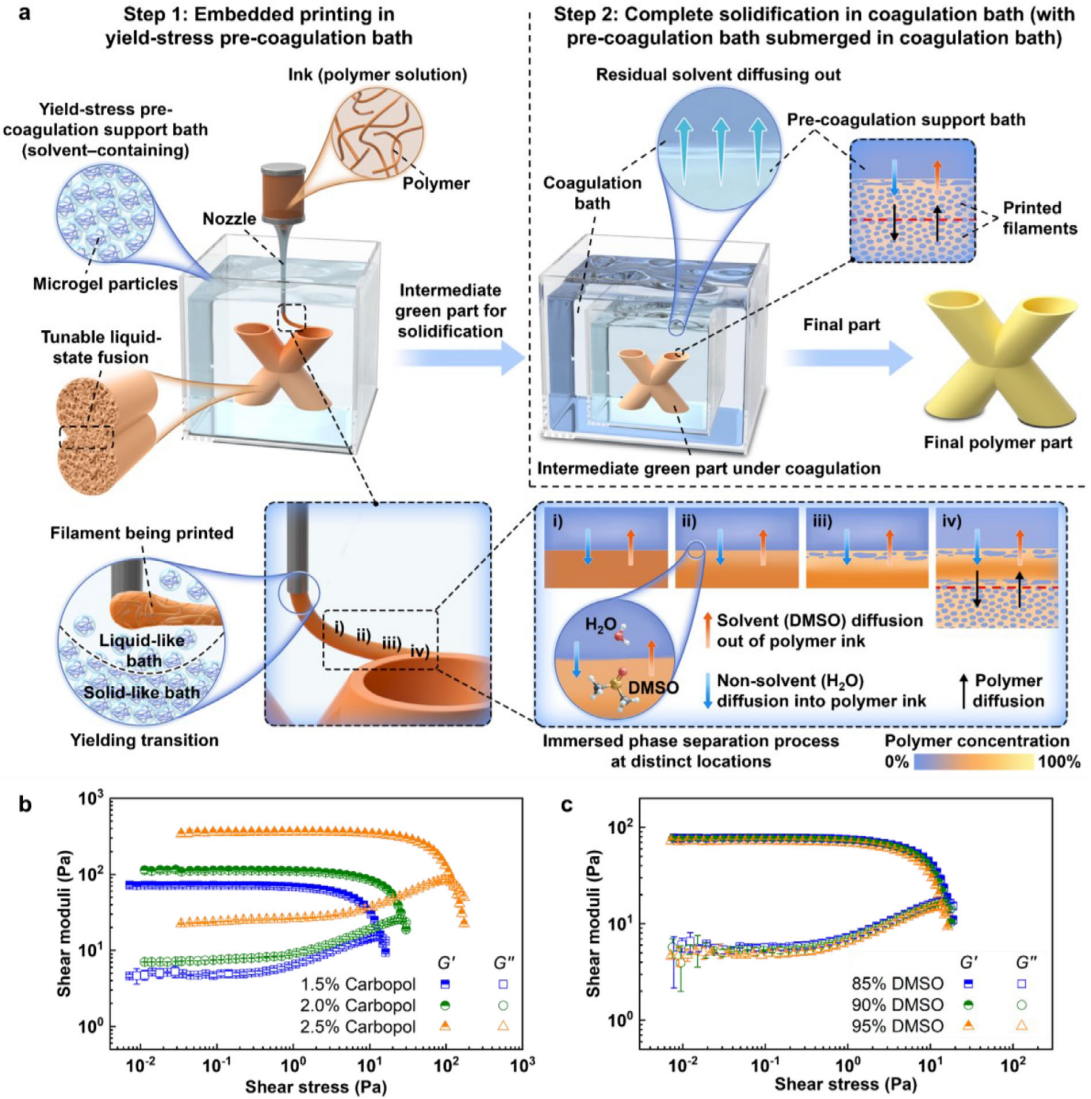
a) Schematic of two‐step IPS‐E3DP process of generic, dissolvable, polymer‐based ink and IPS process. b) Rheological properties of 1.5, 2.0, and 2.5 w/v% Carbopol support bath with 95 v/v% DMSO. c) Rheological properties of 1.5 w/v% Carbopol support bath with 85, 90, and 95 v/v% DMSO.

By utilizing IPS as a solidification and fusion mechanism in embedded 3D printing (E3DP) as a two‐step IPS‐E3DP method, complex 3D structures can be printed using a variety of engineering polymer‐based inks in a solvent‐rich yield‐stress pre‐coagulation support bath (Step 1) and further solidified in a coagulation bath by submerging the pre‐coagulation support bath with the intermediate green part into the coagulation bath, which enables the transport of solvent and non‐solvent at the interface between two baths (Step 2) (**Figure** **2**a). The yield‐stress pre‐coagulation support bath traps the polymer ink in liquid for partial phase separation‐based solidification in a tunable manner. Specifically, the process starts with the printing of a dissolvable polymer‐based ink, which can be made from a single polymer, a polymer blend, or a polymer‐based composite dissolved in a recyclable solvent such as DMSO, in a solvent‐rich non‐solvent (such as water)‐supplemented yield‐stress pre‐coagulation support bath. The bath, containing primarily solvent and a certain ratio of non‐solvent, triggers the IPS process upon contact with the polymer‐based ink and further induces mutual diffusion within the polymer/solvent/non‐solvent ternary system, leading to the gradual segregation of polymer‐rich and polymer‐poor phases. At the same time, the slow phase separation rate of the polymer solution allows a partial polymer‐polymer diffusion between the sequentially deposited polymers (Figure 1a (ii)), resulting in a tunable degree of liquid‐state fusion between them. After the printing, the structure (intermediate green part) embedded in the yield‐stress pre‐coagulation bath and support bath is transferred to a coagulation bath for the polymer‐rich phase to solidify completely (Note S4.1, Supporting Information). Figure 2a (i–iv) details the phase separation behavior at different locations of printed filament during IPS‐E3DP: printing, partial solidification, and fusion in a solvent‐rich yield‐stress pre‐coagulation support bath (Step 1), and complete solidification in a coagulation bath (Step 2). This versatile printing method applies to the printing of any dissolvable engineering polymers and their blends and composites at room temperature. Due to the use of yield‐stress fluid as part of the pre‐coagulation support bath, overhanging and spanning features can be easily printed as well without any support structure, enabling self‐supported printing at the same time (Movie S1, Supporting Information). A detailed comparison of IPS‐E3DP with established additive manufacturing techniques such as fused filament fabrication (FFF), direct ink writing (DIW), and SLA is provided in Note S13 (Supporting Information), highlighting key advantages in material versatility, support‐free printing, and energy efficiency.

Compared with existing printing technologies, IPS‐E3DP excels at self‐supported printing of dissolvable engineering polymers and their derivatives with overhanging and spanning features at room temperature, a capability not currently achievable with conventional printing methods.^[^ ^6^, ^19^, ^20^, ^21^, ^22^, ^30^ ^]^ It is noted that the parts produced often exhibit a porous microstructure, making them particularly useful for applications requiring a large surface‐to‐volume ratio and/or permeability, such as selective filtering and substance storage. Moreover, since IPS‐E3DP is a direct‐ink‐writing process in essence, various materials such as polymer blends, composites, and metals can be easily printed if a dissolvable polymer can be mixed with additives such as short fibers and ceramic or metal powders to make a printable ink. For metal printing, printed intermediate green parts need a post‐printing thermal debinding step to remove the polymer material, which actually functions as a binder during the printing process.

For demonstration, commercially available Carbopol particles were employed as a rheological modifier to endow the pre‐coagulation support bath with suitable yield‐stress rheological properties. Carbopol particles swell in water or other solvents to form microgels, and the jamming of microgels in the solution controls the yield‐stress response^[^ ^26^ ^]^ of the pre‐coagulation support bath. As shown in Figure 2b, the yield stress of the Carbopol pre‐coagulation support bath escalates from ∼10 to 10^2^ Pa as the Carbopol concentration increases from 1.5% to 2.5%. These rheological parameters are elaborated and discussed in Figure S10 and Table S3 (Supporting Information). With the appropriate rheological properties of the Carbopol pre‐coagulation support bath, the moving nozzle partially liquefies the adjacent volume to enable ink deposition, and the pre‐coagulation support bath returns to a solid‐like state (unyielded) after the nozzle leaves to support the deposited ink in situ (Movie S1, Supporting Information). Notably, the addition of different formulations of solvent (such as DMSO) and non‐solvent (such as water) to the Carbopol pre‐coagulation support bath does not significantly influence the much‐needed yield‐stress rheological properties of the bath (Figure 2c).

### 2.3 Engineering Polymer Printing

**Figure 3:**
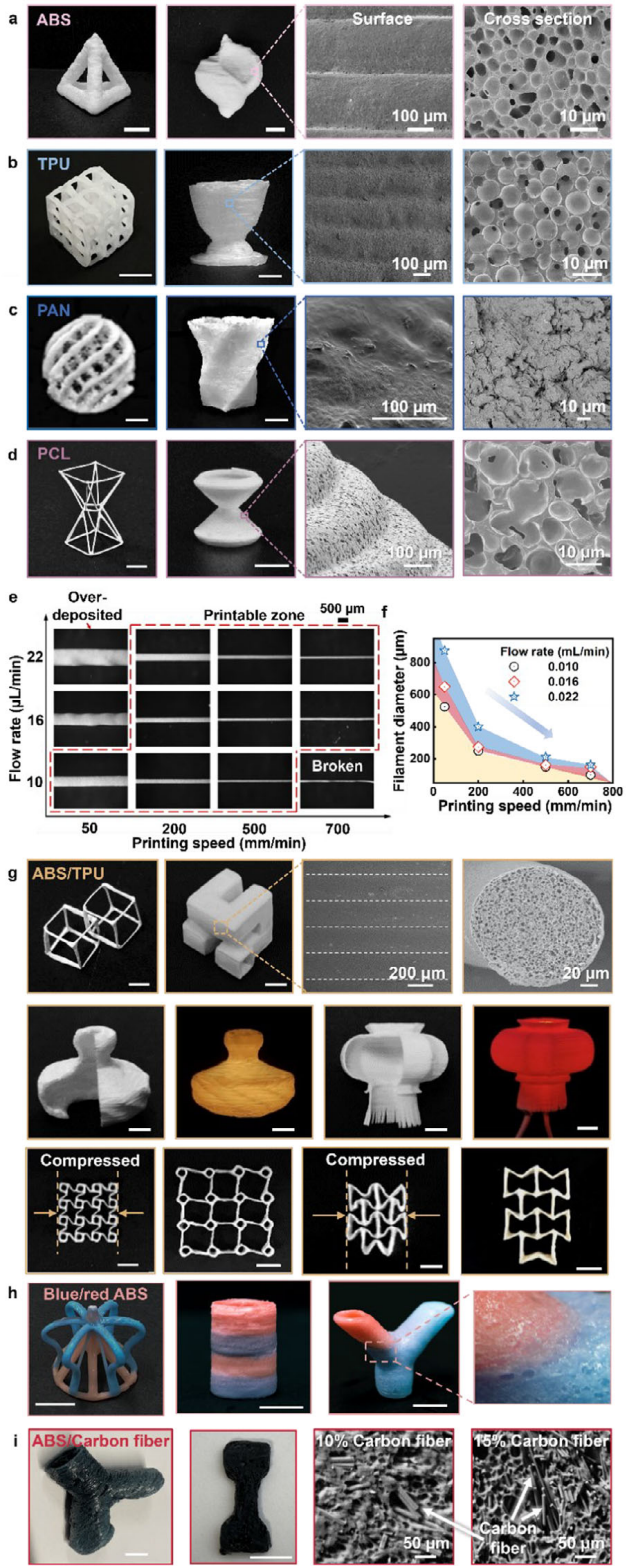
Printed structures, shape fidelity, and their surface and cross‐sectional SEM images. Engineering polymer printing with a) 30 w/v% ABS ink, b) 50 w/v% TPU ink, c) 15 w/v% PAN ink, and d) 27.5 w/v% PCL ink. e) Optical images, and f) diagram of PCL filaments deposited under different printing conditions. g) Hexagonal lasso structure, first‐order Hilbert channel, traditional Chinese lantern structures, forehand structure, and concave hexagon structure printed using polymer blend (ABS (36 w/v%) and TPU (11 w/v%)) ink. Additional polymer printing with h) multi‐material inks, and i) carbon fiber reinforced ABS ink. (Scale bars, if not specified: 5 mm).

To demonstrate the effectiveness and versatility of IPS‐E3DP for producing polymer prints, four typical engineering polymeric materials, namely ABS, TPU, polyacrylonitrile (PAN), and PCL, were utilized to make polymeric inks. Single filament‐based truss structures, as well as solid parts, were printed as shown in **Figure** **3**a–d. Detailed printing parameters, ink formulations, and the rheological properties of inks are summarized in Note S6 (Supporting Information). The solvent selection criteria were based on the polymer solubility as assessed by the relative energy difference (*RED*) parameter, which is based on Hansen's solubility theory (Note S2, Supporting Information). Herein, DMSO was selected as the solvent for these polymers because of its excellent solubility,^[^ ^31^ ^]^ relatively low toxicity, miscibility with water, and recyclability using distillation. Based on the fusion study results, the DMSO concentration in the pre‐coagulation support bath was 95% for ABS ink, 90% for TPU and PAN inks, and 80% for PCL ink.

The printed truss 3D structures, featuring spanning and overhanging features, present substantial challenges for conventional printing technologies that require extensive support to print these elements, if feasible, during fabrication. The first column of Figure 3a–d displays diverse truss configurations, including an ABS tetrahedral frame, a TPU cubic lattice structure, a PAN Christmas ball, and a PCL octadecahedron frame. Although support structures can be incorporated during printing, the aforementioned engineering polymer‐based truss structures are difficult to or cannot be printed without IPS‐E3DP, since it is challenging to print support structures for some spanning and overhanging features, such as the top inverted part of the PCL octadecahedron frame. Successful liquid‐state fusion at junctions of two merging filaments is confirmed by SEM images of the peaks in 3D negative Poisson ratio structures made from PCL (Figure S12a, Supporting Information). The flexibility of printed PCL structures under pressure further highlights the integrity of the printed PCL structure with intricate overhangs and spanning features (Figure S12 and Movies S2 and S3, Supporting Information).

Some solid objects were printed as well to demonstrate the liquid‐state fusion of two adjacent filaments or layers of a surface. The second column of Figure 3a–d illustrates an ABS conch, a TPU egg cup, a PAN helical vase, and a PCL hourglass‐shaped structure, and the third and fourth columns show SEM images of some surfaces and cross sections, respectively, of the solid objects. SEM images of the surfaces confirm good interlayer fusion, and there are no visible cracks or voids between the layers. Cross‐sectional SEM images reveal a uniformly porous internal morphology in the printed parts, resulting from the precisely controlled IPS process. Of the polymers evaluated, PAN exhibits a slower IPS and coagulation process, resulting in decreased porosity. The resulting pore size and mechanical properties are also dependent on the ink formulation (Note S8, Supporting Information). For instance, when the PCL concentration is reduced from 47.1 w/v% to 27.5 w/v%, the average microscopic pore size increases from 5.00 ± 0.47 µm to 8.08 ± 1.13 µm, while Young's modulus decreases from 37.8 ± 4.4 to 22.5 ± 3.6 MPa (both are much lower than that of fused filament fabrication parts (173.3 ± 5.1 MPa)), as discussed in Note S8.1 (Supporting Information). A similar trend is evident with other engineering polymer parts (Notes S8.2 and S8.3, Supporting Information).

Figure 3e,f illustrates that printability is dependent on the extrusion flow rate and printing speed, as during a typical direct ink writing process. Lower flow rates or higher printing speeds produce finer filaments, while higher flow rates or lower printing speeds cause over‐deposition. The dimensional fidelity of printed structures using different polymer inks was selectively analyzed by using *Y*‐shaped tubular structures, showing good dimensional fidelity (Figures S7 and S9, Supporting Information).

### 2.4 Polymer Blend and Multi‐Material Printing

Polymer blends can provide customizable mechanical properties and functionality, which may not be easily printable using fused filament fabrication if they have different melting temperatures. Fortunately, this challenge may not be a concern when using IPS‐E3DP if polymers are dissolvable in the same solvent. For demonstration, the ABS/TPU blend (36 w/v% rigid ABS (melting point of 210–260 °C^[^ ^32^ ^]^) and 11 w/v% flexible TPU (melting point ≈160 °C)^[^ ^33^ ^]^) was prepared for a good balance of strength and flexibility (detailed in Figure S18 and Table S5, Supporting Information). Under a printing speed of 1 mm/s, a resolution of 248.7 ± 4.1 µm and circularity of 0.95 are achieved when printing in a 90% DMSO yield‐stress pre‐coagulation support bath (Figure S4, Supporting Information). Both truss and 3D solid structures were printed using the ABS/TPU blend ink using a single‐nozzle dispenser (Figure 3g; Figures S13 and S14, Supporting Information). The printed hexagonal lasso structure exemplifies the superior fusion at junction points and highlights the full potential of IPS‐E3DP for the polymer blend printing of complex structures. The printed solid first‐order Hilbert channel (Movie S4, Supporting Information) and traditional Chinese lantern structures further demonstrate the resulting high printing quality, good interlayer fusion, and homogenous porous morphology. In addition, the printed ABS/TPU blend auxetic structures (e.g., the forehand structure and concave hexagon) exhibit considerable flexibility, enabling easy deformation and restoration of the original state, and large deformation under tension, expansion, and compression shrinkage (Figure S14, Supporting Information). Since polymer blends can be prepared in situ during printing, functionally graded structures can be printed by incorporating a pre‐printing mixer.

For applications where polymer blends are not expected, individual polymers can be simultaneously printed using multi‐nozzle multi‐material printing to construct a structure. To showcase the multi‐material printing feasibility, blue ABS and red ABS were utilized to print intricate designs such as a complex crown, a straight tube, and a *Y‐*shaped tube (Figure 3h, with the printing process detailed in Movie S5, Supporting Information). The distinct colors delineate structural boundaries but with successful fusion. The intricate crown structure highlights the support‐free precision of the printing process and its potential to create parts with varying properties in different sections, which enables the creation of objects with tailored functionality.

### 2.5 Polymer Composite Printing

Furthermore, the feasibility of polymer composite printing was also investigated when printing ABS and carbon fibers (≈7.2 µm diameter and 100 µm length) through IPS‐E3DP. Figure 3i shows a *Y*‐shaped tubular structure and a dog‐bone specimen made from ABS/carbon fiber. Cross‐sectional SEM analyses reveal uniformly dispersed carbon fibers within the ABS matrix, with short fibers partially aligned axially due to the extrusion‐related shear force. Increasing the carbon fiber content from 0 to 15 w/v% can increase Young's modulus and ultimate tensile strength by 3.52 and 1.56 times, respectively (Figure S19, Supporting Information), providing customizable stiffness and strength essential for 3D sophisticated overhanging structures.

### 2.6 Metal Printing

**Figure 4:**
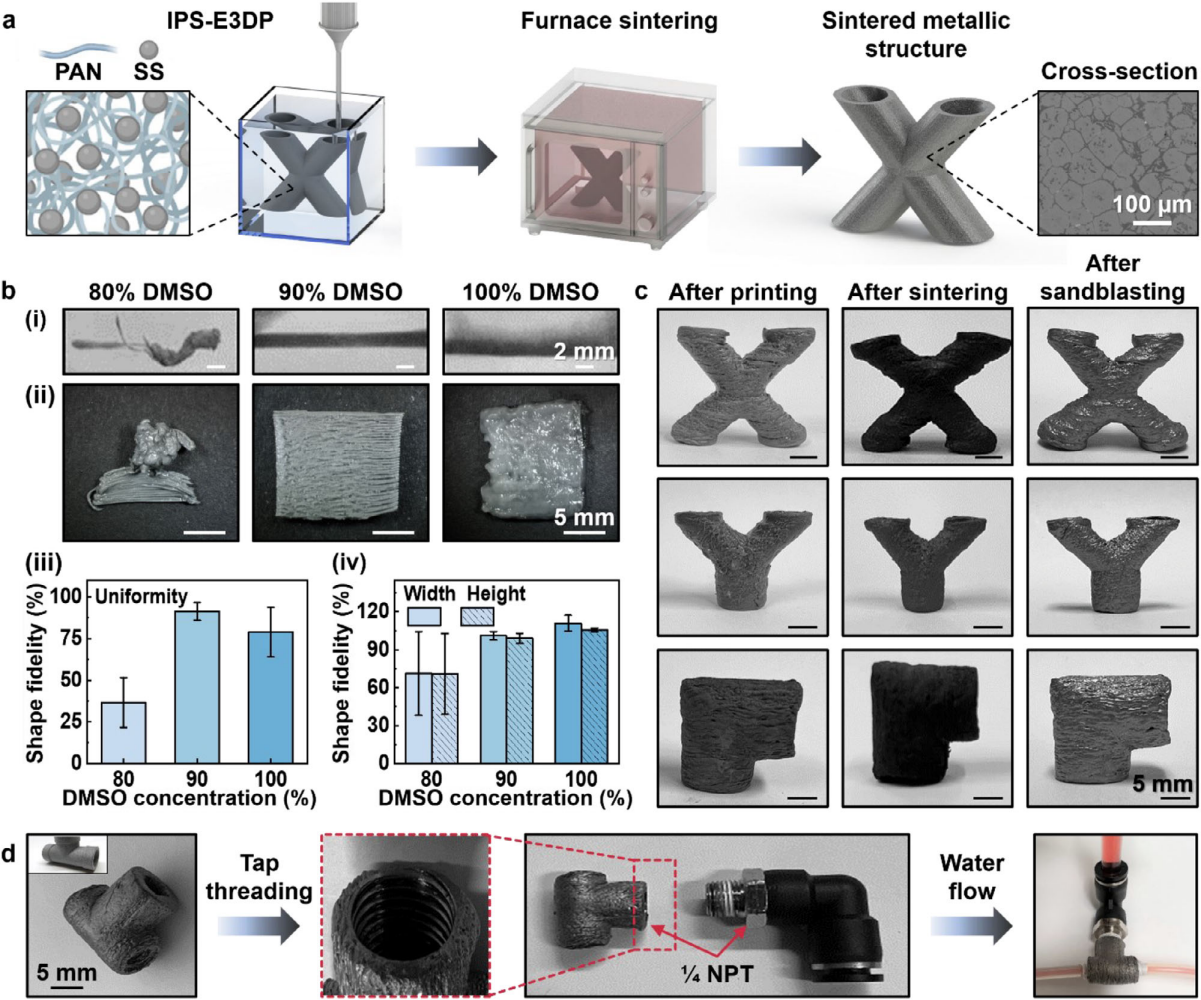
Printing of metal parts. a) Schematic of the metal printing process. b) Liquid‐state fusion study of metal printing based on filaments (i) and sheets (ii) printing, and shape fidelity of filaments (iii) and sheets (iv). c) *X*‐, *Y*‐, and *L*‐shaped stainless‐steel parts after printing, sintering, and sandblasting, respectively. d) Tap threading, assembling, and water flow testing of a printed *T*‐shaped stainless‐steel tube.

If metallic powders are mixed with a polymer solution to make an intermediate green part and the polymer is further thermally removed after printing, the IPS‐E3DP process can provide a complementary approach to printing metallic parts at room temperature. For such an implementation case, the polymer functions as a binder. Briefly, a dissolvable polymer binder‐based metallic intermediate green part is printed first using IPS‐E3DP, followed by thermal debinding to remove the polymer binder and solid‐state sintering to obtain a dense, void‐free metallic structure, as depicted in **Figure** **4**a, reducing material waste and energy cost compared to traditional metal fabrication methods. This study selected PAN as a polymer binder because it undergoes slow coagulation, enabling the fabrication of delicate 3D structures. Stainless steel powder (SS) was chosen as the model metal material, and DMSO/water was used as the solvent/non‐solvent pair. The cross‐sectional SEM image in Figure 4a shows the part microstructure and distinct grain boundaries, indicating the effective removal of the polymer binder (Figure S22, Supporting Information) and the success of the sintering process. It is noted that PAN may not be recommended for some applications since it may still leave some carbide residual after thermal debinding.^[^ ^34^ ^]^ Instead, another polymer, such as ABS, may be used.

As illustrated in Figure 4b, a high solvent concentration in a pre‐coagulation support bath leads to a slower solidification rate and enhances interlayer fusion. When the ink is deposited in a pre‐coagulation support bath with a relatively low solvent concentration, such as 80%, it struggles to maintain its shape and may get dragged away by the dispensing nozzle. In contrast, the ink in a well‐balanced pre‐coagulation support bath retains its shape effectively. It is noted that without non‐solvent, the ink tends to diffuse into the bath (Figure 4b (i), 100% DMSO), leading to distorted filaments during stainless steel‐PAN sheet printing. Both too‐low and too‐high solvent/non‐solvent ratios may result in structures with poor shape fidelity (Figure 4b (ii)). These observations are detailed in the shape fidelity analysis shown in Figures 4b (iii),(iv). Figure 4c displays some printed complex metallic structures, including *X*‐shaped (60° overhang), *Y*‐shaped (45° overhang), and *L*‐shaped (90° overhang) tubular structures with overhang features after printing, after sintering, and after sandblasting, respectively. A detailed analysis of the shape fidelity of these structures is available in Figure S21 (Supporting Information). For demonstration, a printed and sintered *T*‐tube part was subjected to conventional post‐processing steps such as sandblasting for deburring and tap threading for being a piping component (1/8 national pipe thread (NPT)) (Figure 4d) while maintaining the integrity of the thin features (all tubes had a thin wall with ≈1.2 mm thickness). As seen from the leaking test (Figure 4d; Movie S6, Supporting Information), no leakage of the liquid is observed, proving the integrity of the printed stainless‐steel *T*‐tube.

### 2.7 Porous Structure Printing

Porous structures are usually desirable for applications in adsorption, separation, sensing, and biomedical engineering for their significantly large surface area and good permeability. While they can be typically prepared using air injection or porogens during molding or 3D printing, the IPS‐E3DP process uniquely utilizes the IPS as the pore formation mechanism to fabricate 3D homogeneous porous structures. Since no mold making or the use of porogen is involved, IPS‐E3DP can be easily implemented as an efficient and economical alternative for porous structure printing.

**Figure 5:**
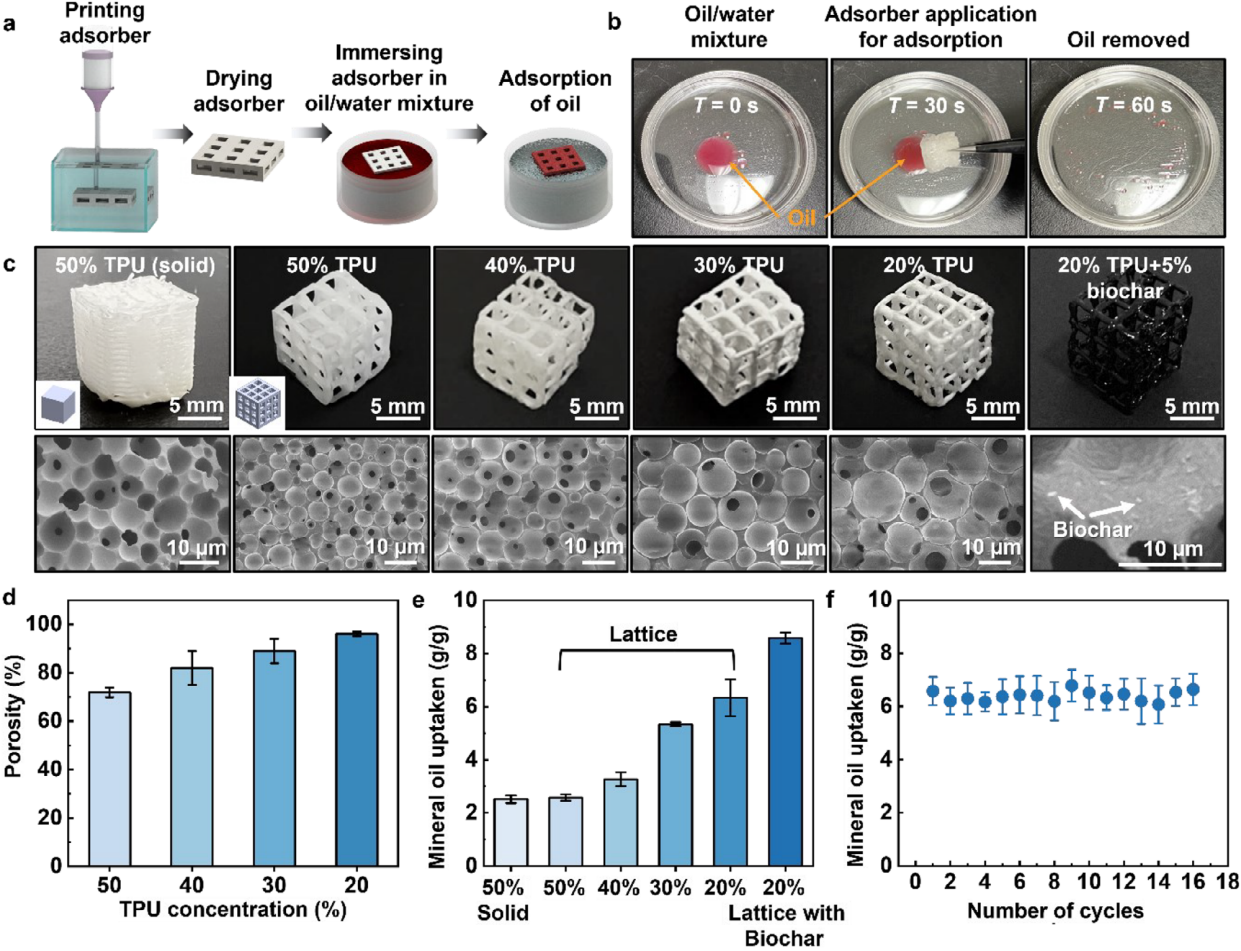
TPU‐based oil adsorber printing and evaluation. a) Schematics of adsorber printing and oil adsorption process. Created with BioRender.com. b) Photographs of the oil adsorption process of TPU adsorbers. c) Printed TPU adsorbers (upper row) and SEM images of intra‐filament pores (lower row). d) Porosity of adsorbers printed with different ink concentrations. e) Oil uptake properties of different printed adsorbers. f) Cycle test of mineral oil uptake.

**Figure** **5**a,b shows the making and testing of TPU‐based porous lattice adsorbers, respectively. For comparison, solid cubes with 50 w/v% TPU and lattice structures with varying TPU concentrations (20, 30, 40, and 50 w/v% TPU) were printed (Figure 5c). Figure 5d shows that the porosity of each lattice adsorbent increases with the decrease of the TPU concentration: 96.08% of adsorbents made from 20 w/v% TPU versus 71.86% of adsorbents made from 50 w/v% TPU. SEM analysis and numerical simulations reveal an inverse relationship between the structural porosity and ink concentration (Figure S2, Supporting Information). Adsorption capacity tests demonstrate that the 20% lattice adsorber achieves the highest equilibrium uptake of 6.33 g/g, significantly surpassing those fabricated with denser TPU inks (for example, 2.58 g/g for 50 w/v% TPU, which is similar to that of the solid cube adsorber (2.52 g g^−1^)) (Figure 5e). Further enhancement in oil adsorption can be seen by integrating biochar into the TPU ink (Figure 5e), leveraging the nanoporous structure of biochar (Figure S23a, Supporting Information) to augment the surface area available for oil molecule attachment. The printed oil adsorber (20 w/v%) demonstrates a comparable oil uptake capacity when compared with that of congeners reported.^[^ ^35^, ^36^ ^]^ Longevity tests further confirm the durability of the adsorption performance, with the oil uptake capacity remaining consistent across sixteen test cycles (Figure 5f). The larger surface area of the lattice adsorbers also significantly reduces the diffusion time and improves the oil transfer efficiency, leading to a relatively rapid kinetic uptake (≈5 s vs 30 s for the solid adsorbent) (Figure S23b, Supporting Information). This study underscores the convenience of the IPS‐E3DP method in making porous structures with varying porosity, paving the way for its application in environmental remediation tasks such as water treatment, air filtration, and oil spill mitigation. The ability to customize the structure porosity, as well as include additional adsorbents such as biochar, further enhances their applicability for a wide range of industrial and environmental applications.

The IPS‐E3DP process can be further implemented to fabricate multi‐scale porous structures with inter‐filament macropores created based on printing conditions such as the filament hatch distance for lattices and intra‐filament micropores generated from the phase separation process. Such multi‐scale porous structures can be utilized for various applications, such as biomineralization using microbially‐induced calcium carbonate (CaCO_3_) precipitation (MICP). Specifically, the macropores create channels that facilitate the diffusion of nutrients, water, and chemicals, and micropores enhance the microorganism attachment and growth due to the increased surface area, which boosts microbial adhesion sites,^[^ ^37^, ^38^ ^]^ and CaCO_3_ deposition.

**Figure 6:**
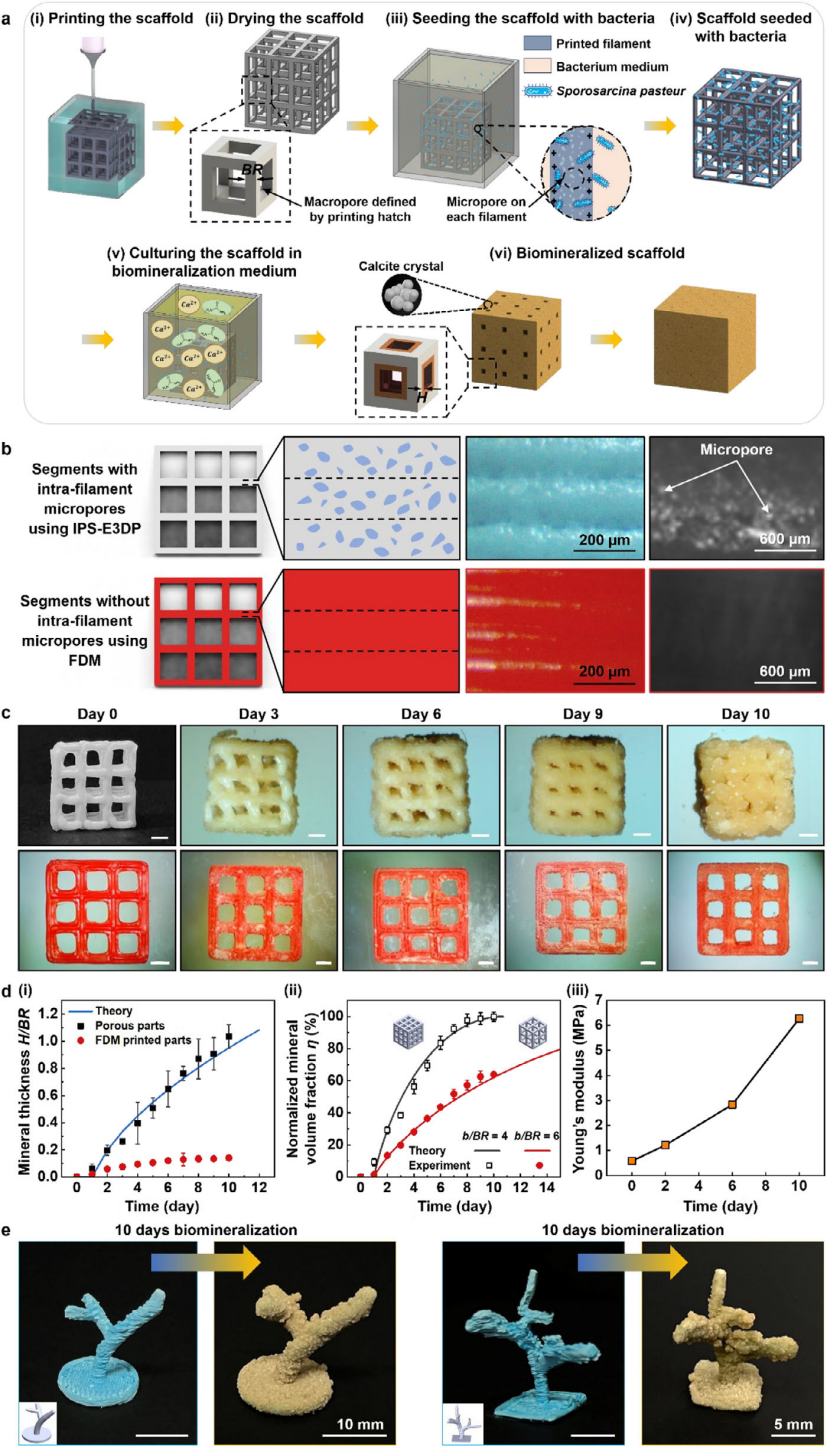
Printing of multi‐scale porous structures. a) Schematics of the multi‐scale porous structure printing and its biomineralization process. b) Schematic and surface microstructure of the printed lattice structure. c) Mineral growth over 10 days. d) (i) normalized mineral thickness and (ii) normalized mineral volume fractions as a function of mineralization time. (iii) Young's modulus of the mineralized samples with different biomineralization times. e) Printed polymer‐based complex coral reef skeletons and biomineralized structures after a ten‐day biomineralization process. (Scale bars: 5 mm if not specified).

For demonstration, 3D multi‐scale porous polymer (ABS) structures were fabricated using the IPS‐E3DP method and biomineralized through the MICP process (**Figure** **6**a). Particularly, a 3 × 3 cubic polymer lattice structure with a beam radius (*BR*) of 0.5 mm and unit cell size (*b*) of 2.0 mm was designed and printed using ABS (Figure 6a–c; Figure S25, Supporting Information) as multi‐scale porous scaffolds. *Sporosarcina pasteurii* was used to mineralize the scaffolds (Figure S24, Supporting Information). For comparison, non‐porous scaffolds were also printed using fused filament fabrication and biomineralized accordingly. While micropores are visible in the segments of the scaffolds, there is no micropore in the scaffolds printed using fused filament fabrication (Figure 6b). Figure 6c shows a significant accumulation of CaCO_3_ minerals (yellow color) occupying most of the vacant spaces over a 10‐day culturing period, and the scaffold surface is tightly covered with minerals. To estimate the MICP rate, the thickness (*H*) of the mineral layer grown around a polymer segment is evaluated. Both the normalized thickness of minerals (*H/BR*) and normalized volume of mineral coverage (*η*) exhibit a continuous growth over 10 days until all the lattice spaces in a scaffold are filled (Figure 6d (i),(ii); Figure S25b, Supporting Information). Notably, the mineralization rate of the IPS‐E3DP printed scaffolds is 7.5 times faster than those produced using fused filament fabrication by calculating the normalized thickness of minerals (*H/BR*) from Figure 6d (i), demonstrating that the 3D multi‐scale porous structure enhances the biomineralization rate. A comparison of the results obtained with the 3 × 3 and 2 × 2 lattice structures indicates that the volume fraction of minerals increases more rapidly as the unit cell size decreases (Figure S25b, Supporting Information). This can be attributed that a smaller unit cell size corresponds to a larger surface area, which promotes the MICP process. Moreover, the effective stiffness of the virgin lattice structure increases from 58.09 to 627.05 MPa after a ten‐day biomineralization period, representing a 10.79‐fold increase (Figure 6d (iii); Figure S26, Supporting Information). More intricate 3D structures can also be printed and fully biomineralized (Figure 6e). The successful biomineralization observed in our scaffold confirms the viability of the embedded bacteria, indicating that the residual DMSO concentration is sufficiently low to avoid cytotoxic effects and supports microbial survival. The high biomineralization rate of multi‐scale porous polymer structures printed using the IPS‐E3DP method highlights the IPS‐E3DP potential for diverse applications in tissue engineering and environmental remediation, where improved nutrient transport and increased surface area are expected.

## 3 Conclusion

This study introduces a two‐step immersion‐phase separation‐based embedded 3D printing (IPS‐E3DP) method for the self‐supported room‐temperature fabrication of a broad array of dissolvable engineering polymers, polymer blends, and polymer composites. IPS‐E3DP is implemented by first depositing polymer inks in a solvent‐rich (typically 80% or higher) yield‐stress pre‐coagulation support bath to have an intermediate green part as the first step and then immersing the pre‐coagulation bath and intermediate green part in a non‐solvent coagulation bath for complete solidification as the second step. The interlayer fusion during embedded printing is a tunable liquid‐state fusion, in essence, as enabled by using a solvent‐rich pre‐coagulation support bath during the first step. The yield‐stress fluid nature of the bath supports a printed structure staying in liquid while enabling self‐supported printing, and the solvent‐rich nature of the bath minimizes the phase separation‐based solidification rate, both ensuring liquid‐state fusion during printing. The IPS‐E3DP facilitates the freeform printing of 3D complicated overhang and/or spanning structures from dissolvable engineering polymer and polymer‐based materials at room temperature. A variety of 3D engineering polymer and/or polymer‐based structures are successfully printed to demonstrate the versatility and capability of IPS‐E3DP. Furthermore, IPS‐E3DP can be utilized to print metals by using polymers as thermally removable binders in addition to 3D porous structures. It is anticipated that this printing method offers significant advancements in producing 3D hierarchical, porous objects with diverse applications related to polymer printing, metal printing, and environmental protection, to name a few.

## 4 Experimental Section

### Ink Preparation

Ink formulations for single polymer printing were created by dissolving the polymers in DMSO (Bioreagent grade, Fisher, Fair Lawn, NJ, USA). This process was facilitated by the use of a heating machine at 80 °C with continuous magnetic stirring at 300 rpm, which was sustained for a period of 1–2 days to ensure the complete dissolution of the polymer in DMSO. For the ABS ink, ABS filaments (ABS pro, Flashforge, Jianhua, Zhejiang, China) were dissolved in DMSO with ABS concentrations of 15.0, 20.0, and 30.0 w/v%. To prepare the recycled ABS ink, the ABS from failed or redundant printed parts was dissolved in DMSO to make a 30.0 w/v% ink. The single TPU ink was prepared by blending 5.0 g of soft TPU (Elastollan soft 35A, BASF, Wyandotte, MI, USA) with 10.0 mL of DMSO, resulting in a nominal concentration of 50.0 w/v% ink. TPU inks with concentrations of 20.0, 30.0, and 40.0 w/v% were also formulated by mixing 2.0, 3.0, and 4.0 g of TPU with 10.0 mL of DMSO, respectively. Additionally, PAN ink (150 kDa, Pfaltz and Bauer, Waterbury, CT, USA) was dissolved in DMSO to create a 15.0 w/v% concentration. PCL inks were prepared by dissolving PCL granules (Mn80000, Macklin, Shanghai, China) at concentrations of either 27.5 or 47.1 w/v% in DMSO.

To formulate the ABS/TPU blend ink, ABS and TPU (65A) were dissolved in DMSO at concentrations of 36.0 w/v% and 11.0 w/v%, respectively, and stirred at room temperature for at least 24 h to ensure thorough mixing. For the fabrication of composite materials, various additives were incorporated into the base polymer inks. For the ABS/carbon fiber composite ink, carbon fibers with a diameter of 7.2 µm and a length of 100 µm (PX35, ZOLTEK, Bridgton, MO, USA) were added in concentrations of 5, 10, or 15 w/v% to the 30 w/v% ABS ink and thoroughly blended using an AR‐100 mixer (Thinky, Laguna Hills, CA, USA) at 2000 rpm for 15 min. In the preparation of metallic composite ink, stainless steel powders (100 mesh, Type 316‐L, Alfa Aesar, Tewksbury, MA, USA) were combined with 15 w/v% PAN ink in a ratio of 5:1 to produce the ink suitable for printing metal parts. For metal part printing, stainless steel powders (100 mesh, Type 316‐L, Alfa Aesar, Tewksbury, MA, USA) were mixed with 15 w/v% PAN ink in a weight ratio of 5:1 to obtain the metallic composite ink. To prepare the TPU‐based oil adsorber with biochar, 5 w/v% biochar (Black Owl biochar, Biochar Supreme, Everson, WA, USA) was mixed with TPU using a mixer at 2000 rpm for 15 min. The ink formulations are listed in Table S4 (Supporting Information).

### Preparation of Support Bath

To formulate a 2.5 w/v% Carbopol pre‐coagulation support bath with 90% DMSO, 45 mL of DMSO and 5 mL of deionized water were combined to achieve a 9:1 DMSO: water ratio, totaling 50 mL. Subsequently, 1.25 g of Carbopol 940 (Lubrizol, Cleveland, OH, USA) was added, mixed thoroughly until homogeneous, and then left to stabilize for a minimum of 12 h. Similarly, Carbopol solutions at concentrations of 1.5, 2.0, and 3.0 w/v% were prepared by adjusting the Carbopol mass fraction. The DMSO content in these solutions varied from 0 to 100 v/v% by manipulating the DMSO: water ratio.

### Deposition/Printing Procedure

3D printing was performed using a Hyrel Engine SR printer (Hyrel3D, Norcross, GA, USA) with a CSD‐5 dispensing head (the UV array was removed for this study) controlled by the Repetrel software interface. The ink was loaded into a disposable 5 mL syringe fitted with a stainless‐steel tip (Nordson EFD, Vilters, Switzerland). The STL models required for the printing process were sliced using the Slic3r utility embedded within Hyrel's Repetrel software. This slicing process created G‐code files that were then used to control the printer. Comprehensive details on the printing parameters, including nozzle diameter, printing speed, and extrusion flow rate for each printing experiment, are provided in Table S4 (Supporting Information).

### Post‐Deposition/Printing Solidification in Non‐Solvent‐Only Coagulation Bath

Following the completion of a printing process, the support bath was fully submerged in water as a whole for an entire night (Figure 2a; Figure S8, Supporting Information). This was done to substitute the solvent (DMSO) in the support bath with a non‐solvent (water). In this way, the printed parts within the support bath were allowed to coagulate (Note S4.1, Supporting Information). Subsequently, the support bath material was removed from the solid object that had been printed.

### Debinding and Sintering

The printed intermediate green structures were successfully debinded and sintered in a tube furnace oven. The 3D‐printed samples were heat‐treated in an alumina tube furnace (VTF‐1700, Zylab, China) and placed in a magnesium or aluminum crucible. The debinding and sintering processes were performed under vacuum conditions using a rotary vane vacuum pump (EQ‐FYP, MTI Corp, Richmond, CA, USA). The temperature profile of the furnace is illustrated in Figure S20 (Supporting Information).

### Material Characterization—*Rheology Measurements*

The analysis of rheological characteristics was carried out using a rheometer (MCR‐702 TwinDrive, Anton‐Paar, Graz, Austria). The device utilized a 25 mm sandblasted parallel plate measuring geometry with a roughness average (*R_a_*) of 4.75 µm and a 1 mm gap. Steady‐rate sweeps were conducted by varying the shear rate from 100 to 0.01 s^−1^. To mitigate loading effects, a pre‐shear step was incorporated at 100 s^−1^ for 30 s, and then a recovery period of 1 min was given to allow the structure to restore itself. Additionally, a strain sweep was performed within a range of 1–100% strain at a low frequency of 1 Hz.

### Material Characterization—*Mechanical Tests*

The mechanical properties of the printed polymer and composite parts were measured using a microtester (eXpert 4000, Admet, Norwood, NA, USA). All the tests involving tension and compression were conducted with a jog rate set at 0.5 mm s^−1^. The data corresponding to load was gathered utilizing a load sensor with a capacity of 1000 g, and the process was halted when it reached 90% of the peak load. The stress–strain relationship was plotted based on the load, displacement, and sample shape. The Young's modulus of each sample was calculated by the linear portion of the stress–strain plot.

### Material Characterization—*Imaging (SEM)*

The microstructures of the 3D‐printed items were characterized by SEM (S‐3000, Hitachi, Ibaraki, Japan or GeminiSEM 300, ZEISS, Germany). Prior to the SEM analysis, the samples underwent a coating process with a 10 nm‐thick gold‐palladium (Au‐Pd) layer.

### Material Characterization—*Pore Size and Porosity*

To measure the pore size and porosity of the samples, the ImageJ software (NIH, Bethesda, MD, USA) was employed on the cross‐sectional SEM images. The images were converted to 8‐bit grayscale, and the threshold was adjusted to distinguish the pores from the matrix clearly. The “Analyze Particles” tool measured the size and distribution of the pores, with size and circularity parameters set to filter out noise. The images were converted to binary format for porosity calculation, and the “Analyze Particles” function quantified the pore area relative to the total image area.

### Material Characterization—*Adsorber Measurement*

All the printed adsorbents were subjected to an adsorption capacity test. To perform this test, the mass of each adsorber was measured using an analytical balance (ML54, Mettler Toledo, Columbus, OH, USA). Thereafter, each adsorber was fully submerged in mineral oil for exactly 1 min. The timer started when the adsorber came into contact with the oil. After 1 min, the adsorber was removed and immediately weighed using an analytical balance to obtain the final mass. For the cyclic adsorption tests, excess mineral oil on the surface of the adsorber was carefully blotted off after each 1‐min immersion before proceeding to the next cycle. The adsorption capacity of each adsorber was then calculated according to Equation (2):

(2) $$c=\frac{m_{end}-m_{begin}}{m_{begin}}$$

where *c* is the adsorption capacity in grams of oil per gram of adsorber, *m_end_* is the final mass of the adsorber immediately after removal from the oil, and *m_begin_* is the initial mass of the adsorber.

### Biomineralization Process

A biomineralization medium known as BPU medium (ATCC 1832 medium) was prepared, containing 10 g L^−1^ tryptone, 5 g L^−1^ yeast extract, 4.5 g L^−1^ tricine, 5 g L^−1^ ammonium sulfate, 2 g L^−1^ glutamic acid, and 10 g L^−1^ urea. The pH was adjusted to 8.6 ± 0.1 with a NaOH solution and then sterilized by passing through a 0.2 µm sterile filter. A mineralization medium was also prepared by mixing 3 g Difco nutrient broth, 20 g urea, 10 g ammonium chloride, and 2.12 g sodium bicarbonate in 1 L of deionized water. The pH was reduced to below 6.0 before sterilizing the medium by autoclaving at 121 °C for 45 min. After cooling, 28 g of CaCl_2_ was added to this sterile solution. The 3D printed samples, after drying, were submerged in a BPU medium containing bacteria and incubated at 30 °C for 24 h. These structures were then soaked in the mineralization medium, placed in an incubator set to 28 °C, and stirred at a speed of 500 rpm. This medium was replaced on a daily basis. After the biomineralization process, the samples were removed and cleaned with a 70% alcohol solution.

## Conflict of Interest

The authors (K.S., A.M.C., Y.H.) declare competing financial interests with the two issued patents: Methods and Apparatuses for Freeform Additive Manufacturing of Engineering Polymers, Huang, Y., Compaan, A.M., and Song, K., US Patent 10,974,441 B2, April 13, 2021 and U.S. Patent 11,724,440 B2, August 15, 2023.

## Author Contributions

K.S., Q.W., and A.M.C. equally contributed to this work. K.S., Q.W., A.M.C., Y.H., and J.Y. planned and designed the project. K.S., Q.W., A.M.C., J.S., C.Z., and M.C. prepared the materials and conducted the experiments. K.S., Q.W., A.M.C., J.S., and B.R. collected and analyzed data. K.S., Q.W., A.M.C., J.S., C.Z., M.S.G., and J.T.R. characterized the samples. K.S., Q.W., and A.M.C. wrote the manuscript. H.Y., Y.H., and J.Y. directed the writing of the paper. Y.H. and J.Y. supervised the project.

## Supporting information



Supporting Information

Supplemental Movie 1

Supplemental Movie 2

Supplemental Movie 3

Supplemental Movie 4

Supplemental Movie 5

Supplemental Movie 6

## Data Availability

The data that support the findings of this study are available from the corresponding author upon reasonable request.
