# Supplementary material for: Solvent‐Rich Pre‐Coagulation Bath for Tunable Liquid‐State Fusion Enables Robust Two‐Step Polymer Embedded Printing
Source: Adv Sci (Weinh). 2025 Jul 28;12(40):e08335. doi: 10.1002/advs.202508335 (PMC12561439; doi:10.1002/advs.202508335)
Supplement: Supplementary file 1 — Supporting Information [file ADVS-12-e08335-s006.pdf]

Supplementary Information for:

Solvent-Rich Pre-Coagulation Bath for Tunable Liquid-State Fusion Enables Robust Two-Step Polymer Embedded Printing

Kaidong Song[+], Qian Wu[+], Ashley M. Compaan[+], Junting Shen, Chuanshen Zhou, Mingwei Chen, Marc Sole-Gras, John-Thomas T. Robinson, Bing Ren, Huayong Yang, Yong Huang & Jun Yin**

([+]These authors made equal contributions)

K. Song, C. Zhou, M. Sole-Gras, B. Ren, Y. Huang

Department of Mechanical and Aerospace Engineering

University of Florida

Gainesville, FL 32611, USA

E-mail: yongh@ufl.edu

Q. Wu, J. Shen, M. Chen, H. Yang, J. Yin

The State Key Laboratory of Fluid Power and Mechatronic Systems, School of Mechanical Engineering

Zhejiang University

Hangzhou, 310028, China

E-mail: junyin@zju.edu.cn

A. M. Compaan, J. T. Robinson, Y. Huang

Department of Materials Science and Engineering

University of Florida

Gainesville, FL 32611, USA

E-mail: yongh@ufl.edu

Supplementary Note 1. Phase separation and fusion of polymer solution

In immersion phase separation (IPS) studies, the ternary phase diagram of a polymer/solvent/non-solvent system is essential for predicting the behavior of mixtures under varied compositions. A prime example is the ternary phase diagram for the thermoplastic polyurethane (TPU)/dimethyl sulfoxide (DMSO)/water system (**Figure S1a**), determined based on the extended Flory–Huggins theory, which provides critical thermodynamic insights into the phase separation process.

For a ternary system, there is:

$$\varphi_n + \varphi_s + \varphi_p = 1 \tag{S1-1}$$

where φ_i is the volume fraction of each component (*n*, non-solvent; *s*, solvent; *p*, polymer). As φ_s and φ_p are considered to be independent variables. The ternary Cahn–Hilliard equations[1,2] were used to simulate both diffusion and liquid–liquid phase separation, the non-dimensionalized form of Cahn–Hilliard equation can be expressed as the follows:

$$\frac{\partial \varphi_s}{\partial \tilde{t}} = \tilde{\nabla} \cdot \left(M\_ss\tilde{\nabla}\tilde{\mu}_s + M\_sp\tilde{\nabla}\tilde{\mu}_p\right) \tag{S1-2}$$

$$\frac{\partial \varphi_p}{\partial \tilde{t}} = \tilde{\nabla} \cdot \left(M\_ps\tilde{\nabla}\tilde{\mu}_s + M\_pp\tilde{\nabla}\tilde{\mu}_p\right) \tag{S1-3}$$

where M_ij are the mobilities, $\tilde{\mu}_j$ are the generalized chemical potentials. The system's free energy accounts for interaction parameters (χ_{ij}) and component behaviors which is described by the Flory–Huggins theory[3,4] (S1-4):

$$\frac{\Delta G_m}{RT} = \left(\sum \frac{\phi_i}{m_i} ln\phi_i + \sum \chi_{ij}\phi_i\phi_j\right)\sum m_i n_i \tag{S1-4}$$

Here, *R* and *T* respectively represent the universal gas constant (J/(mol·K)) and the absolute temperature (K), n_i and ϕ_i respectively represent the mole number and the volume fraction of component *i*.

The binodal curve is experimentally determined via cloud-point titration, while the spinodal curve involves calculations from the Gibbs free energy derivatives:

$$\frac{G_{ss}}{RT} = \frac{1}{RT}\frac{\partial^2 \Delta G_v}{\partial {\varphi_s}^2} = \frac{1}{\varphi_s} + \frac{1}{\varphi_n} - 2\chi_{ns} \tag{S1-5}$$

$$\frac{G_{pp}}{RT} = \frac{1}{RT}\frac{\partial^2 \Delta G_v}{\partial {\varphi_p}^2} = \frac{1}{\varphi_n} + \frac{1}{m_p\varphi_p} - 2\chi_{np} \tag{S1-6}$$

$$\frac{G_{sp}}{RT} = \frac{1}{RT}\frac{\partial^2 \Delta G_v}{\partial \varphi_s \partial \varphi_p} = \frac{1}{\varphi_n} - \chi_{ns} - \chi_{np} + \chi_{sp} \tag{S1-7}$$

Determining the χ_{ij}, especially using methods like equilibrium swelling, enables precise fitting of both binodal and spinodal curves, crucial for understanding and predicting the phase behavior in such complex systems.

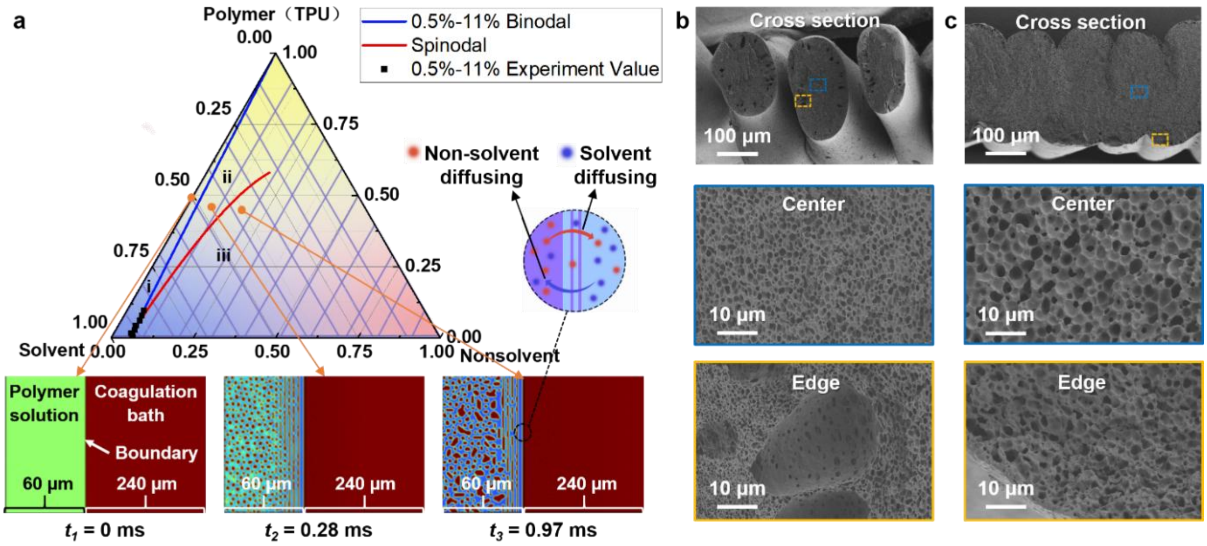

Figure S1. a, Ternary phase diagram and numerical simulation of the phase separation process. **b**, SEM images of solidified polymer cross-section in a solvent-free coagulation bath. **c**, SEM images of solidified polymer cross-section in a solvent-rich coagulation bath.

The Cahn–Hilliard equations were discretized using the finite element method on a regular grid to simulate the TPU/DMSO/water membrane system. To maintain computational efficiency and simplify calculations, the molecular weight and interaction parameters of TPU were adjusted, keeping the spinodal relatively constant. The simulation parameters are provided in **Supplementary Table 1**.

Supplementary Table 1. Parameters for Cahn–Hilliard simulation of the TPU system

m_p	M_ss	M_pp	M_sp & M_ps	K_ss & K_pp	K_sp & K_ps	χ'_{ns}	χ'_{np}	χ'_{sp}
5	2000	2	0	1.6e-5	0	-0.44	5	0.4

Zero-flux boundary conditions were applied in the x direction, and periodic boundary conditions in the y direction. The initial polymer solution layers had varying concentrations φ_{p1} = 0.28 ± 0.005, φ_{s1} = 0.67 ± 0.005, φ_{p2} = 0.38 ± 0.005, φ_{s2} = 0.57 ± 0.005, and φ_{p3} = 0.48 ± 0.005, φ_{s3} = 0.47 ± 0.005). In the coagulation bath, a constant ratio was maintained (φ_p = 0.01 ± 0.005, φ_s = 0.90 ± 0.005[1 - (x - 0.6)] (0.6 ≤ x ≤ 1.4) and 0.90 ± 0.005 * 0.2 (x > 1.4). This ratio represented a yield-stress pre-coagulation support bath with 90% DMSO and 10% water for 0.6 ≤ x ≤ 1.4, and transitioning to a bath of only water for 1.4 ≤ x ≤ 3. The ratio of x dimension to y dimension in the simulation was 3:1, and the grid size was 450 × 150.

The morphological evolution of a 50% TPU solution in a 90% DMSO coagulation bath was numerically simulated, as detailed in **Figure S1a**. Initially, the configuration is bifurcated, with the polymer solution on the left and the bath on the right. At the interface, a dense, porous skin-like layer forms due to phase separation. Despite rapid inter-diffusion of solvent and non-solvent, the polymer mainly stays within the solution area because of a free energy barrier and low mobility. As diffusion continues, the composition reaches the spinodal curve, initiating

phase separation from right to left in a layered structure and leading to spinodal decomposition throughout the layer. This process results in a differentiation into finer polymer-poor and polymer-rich phases, with the polymer-poor phase becoming porous. The solvent concentration significantly impacts the IPS process by affecting phase separation timing, allowing polymer chains more time to rearrange and leading to uniform pore formation. This effect is enhanced in solvent-rich baths compared to solvent-free conditions, confirmed by SEM images (**Figures S1b** and **S1c**).

Figure S2 further illustrates that increasing TPU concentration from 30% to 50% leads to smaller pores, aligning with simulation results. The final TPU membrane morphology features an asymmetric structure with a skin layer over a uniformly porous layer, consistent with experimental observations.

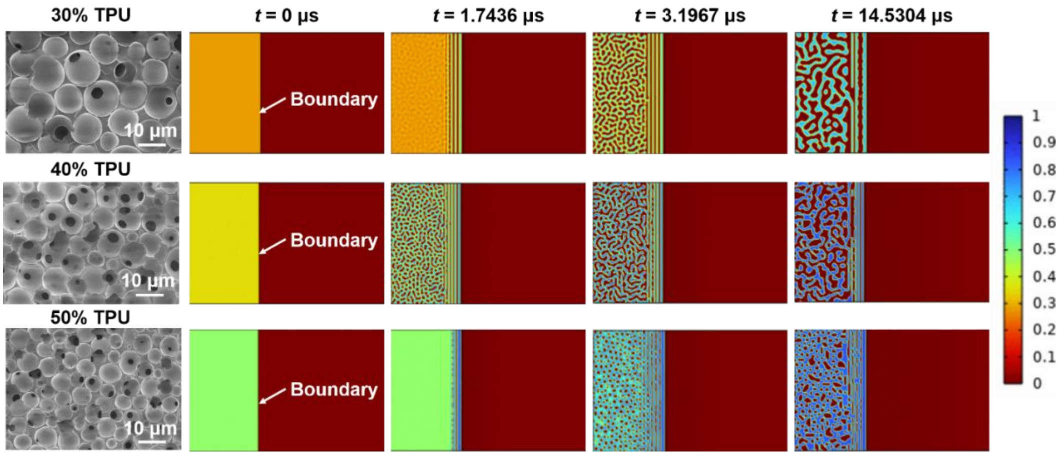

Figure S2. Cross-sectional morphology and simulated morphological evolution process of TPU inks with varying concentrations.

Numerical simulations were performed using COMSOL Multiphysics (Version 6.1, COMSOL Inc., Sweden) to model the inter-diffusion of two polymer solutions. A 2D model with two squares representing the polymers, with initial concentrations of 0 and 1 mol/m^3, simulated diffusion based on Fick's law. No-flux boundaries were applied to prevent external exchange, and the domain was finely meshed with 12,564 elements for precision. Simulations ran over 8 seconds with a 0.01-second timestep, considering diffusion coefficients of 0 and 5E-8 m^2/s to explore a range of diffusion behaviors.

Figure S3 illustrates representative simulation curves for the fusion of polymers *a* and *b*. The process begins upon surface contact, initiating inter-molecular diffusion that causes polymer chain segments to intertwine at the interface. This interaction often leads to entanglements and, potentially, chemical bonds. The efficiency of this process is influenced by the miscibility of the polymers and their molecular compatibility. The strength of the resultant interfacial bonding correlates with the bonding depth (detailed in **Figure S3b**), which is governed by the time-dependent diffusion coefficient, D_{ba}. The simulations show that an increase in D_{ba} enhances the bonding depth over time, highlighting the importance of managing the diffusion coefficient or solvent concentration to optimize liquid-state polymer fusion efficiency in IPS.

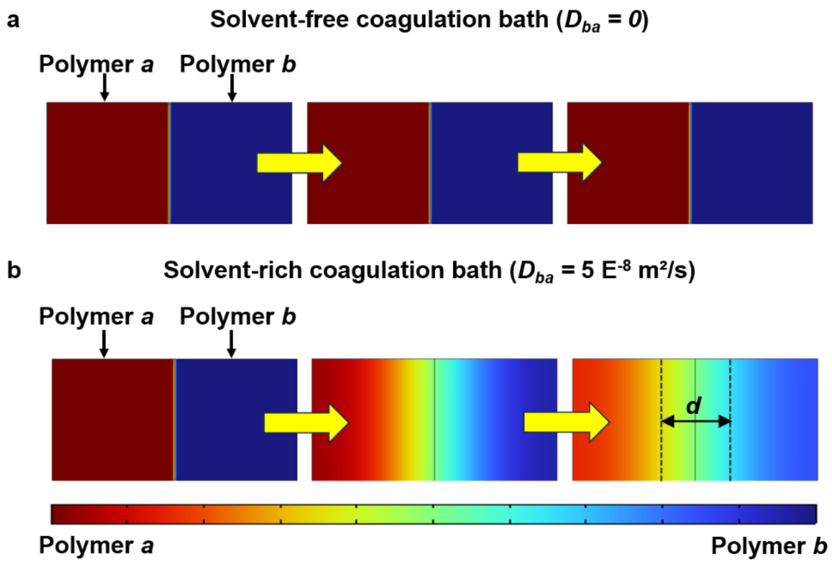

Figure S3. Simulation of polymer-polymer diffusion process: a, solvent-free versus b, solvent-rich coagulation baths.

Supplementary Note 2. Hansen solubility analysis

In order to achieve proper dissolution of a polymer, it is necessary to consider its relative energy difference (*RED*)[5] parameter with respect to the solvent used. For effective dissolution, the *RED* value of the polymer candidate should be lower than 1. This criterion is based on Hansen's chemical affinity[1] relationships, as expressed in Supplementary Equations S2-1 and S2-2:

$$R_a^2 = 4(\delta_{d2} - \delta_{d1})^2 + (\delta_{p2} - \delta_{p1})^2 + (\delta_{h2} - \delta_{h1})^2 \quad \text{(S2-1)}$$

$$RED = \frac{r_a}{r_0} \quad \text{(S2-2)}$$

The δ_d, δ_p, and δ_h parameters represent the energy from dispersion forces, dipolar intermolecular forces, and hydrogen bonds between molecules, respectively, all in units of $MPa^{0.5}$. r_a is the distance in the Hansen space, and r_0 is the interaction radius. Energy values for each parameter are obtained from published literature, and the calculated *RED* values for acrylonitrile butadiene styrene (ABS), TPU, polyacrylonitrile (PAN), and polycaprolactone (PCL) are listed in **Supplementary Table 2**, demonstrating that these polymers can be dissolved using DMSO.

Supplementary Table 2. *RED* calculations

Component	δ_d	δ_p	δ_h	r_0	*RED*
DMSO[5]	18.4	16.4	10.2	/	/
ABS[6]	17.6	8.6	6.4	10.9	0.81
TPU[7]	18.8	10	8.2	9.8	0.68
PAN[5]	21.7	14.1	9.1	10.9	0.65
PCL[8]				11.9	0.87

Supplementary Note 3. Effect of DMSO concentration on shape fidelity during solidification and fusion

This study examines how solvent extraction affects polymer solidification or coagulation. A yield-stress pre-coagulation support bath formulated with DMSO as the solvent and water as the non-solvent promotes gradual coagulation by facilitating solvent diffusion from the polymer into the bath. This approach ensures consistent quality across various printed formats, including filaments, films, and 3D objects.

Supplementary Note 3.1. Filament morphology

Filament morphology, highly dependent on the solidification rate, is analyzed using ABS/TPU ink in **Figure S4**. At a printing speed of 1 mm/s, filaments in a 90% DMSO bath maintain their intended diameter (~270 μm) and rounded shape, while those in 70% and 80% DMSO baths exhibit significant shrinkage and less circular cross-sections due to faster solidification, highlighting how solvent concentration affects print quality.

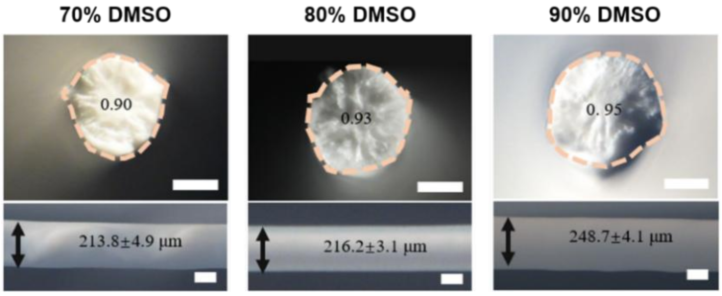

Figure S4. Printed ABS/TPU filament analysis.

Supplementary Note 3.2. Film printing

This study evaluated the impact of various DMSO concentrations in the yield-stress pre-coagulation support bath on the fusion processes and shape fidelity of films (15 mm × 15 mm) made from single ABS ink and ABS/TPU composite ink.

For the single ABS ink, **Figure S5** illustrates that higher DMSO concentrations enhance surface smoothness and inter-filament bonding, crucial for structural integrity. These improvements result from slower solidification rates that facilitate better filament fusion. The shape fidelity was evaluated by measuring actual dimensions against intended designs, confirming that increased DMSO concentrations improve dimensional accuracy.

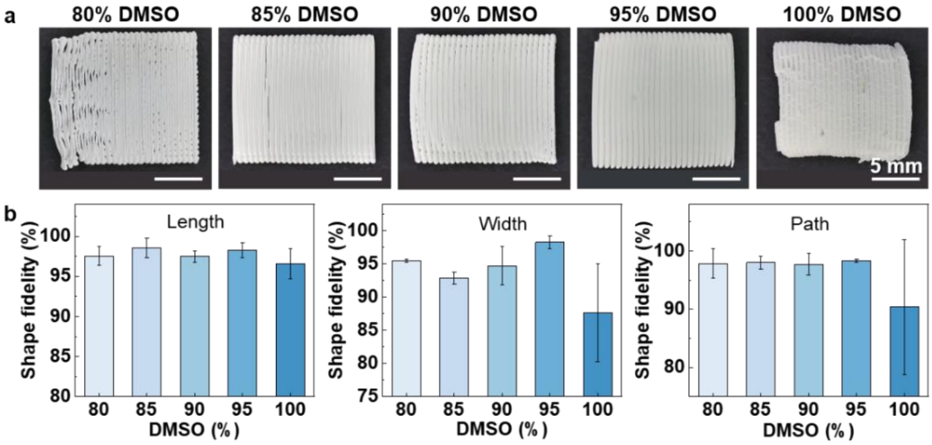

Figure S5. Printed ABS film analysis.

For the ABS/TPU composite ink, films were made with DMSO concentrations from 50% to 100%, documented in **Figure S6**. At 50% DMSO, rapid solidification resulted in poor shape fidelity and visible filament separation. Conversely, higher DMSO concentrations (70% and above) slowed solidification, enhancing polymer diffusion and filament fusion. The optimal concentration was found to be 90% DMSO, which aligned the modulus of the ink with the support bath, facilitating effective interactions and uniform polymer phase distribution. At 100% DMSO, excessive polymer diffusion caused fuzzy film surfaces, indicating overly high concentration.

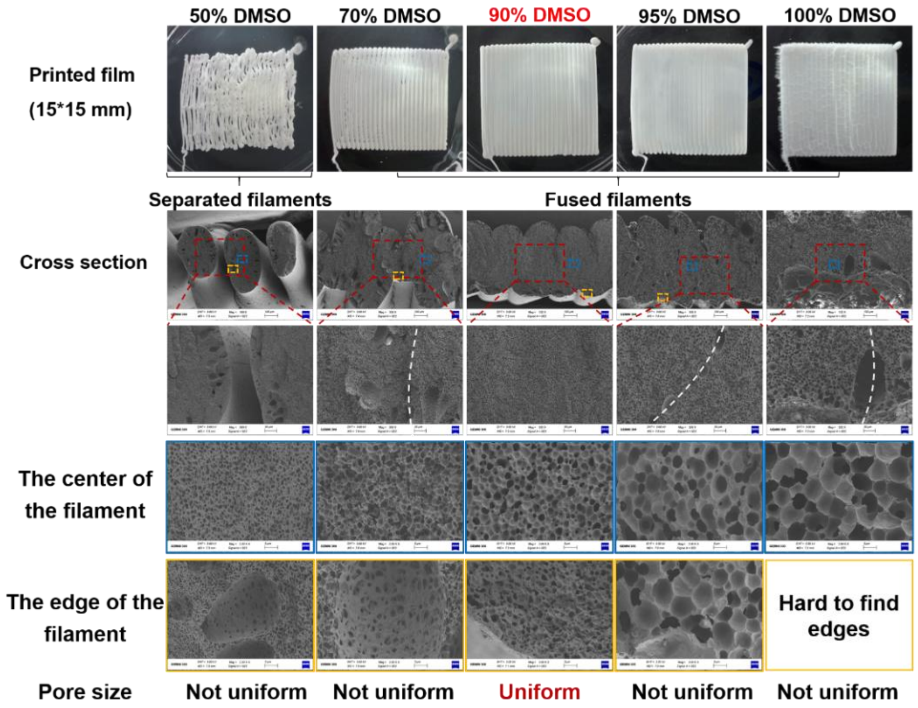

Figure S6. Printed ABS/TPU film analysis.

Supplementary Note 3.3. 3D tube printing

Filament fusion significantly impacts the overall structural integrity of the final 3D-printed object. The shape fidelity was analyzed with printed 3D tubular-shaped structures.

For TPU ink, results are reasonably similar for yield-stress pre-coagulation support bath material formulations with 85% or higher DMSO content in the yield-stress pre-coagulation support bath material, as shown in **Figure S7a**. Like TPU ink, PAN-based ink is less sensitive to the yield-stress pre-coagulation support bath material composition and yield-stress pre-coagulation support bath material formulations with 90% or higher DMSO content is selected (**Figure S7b**).

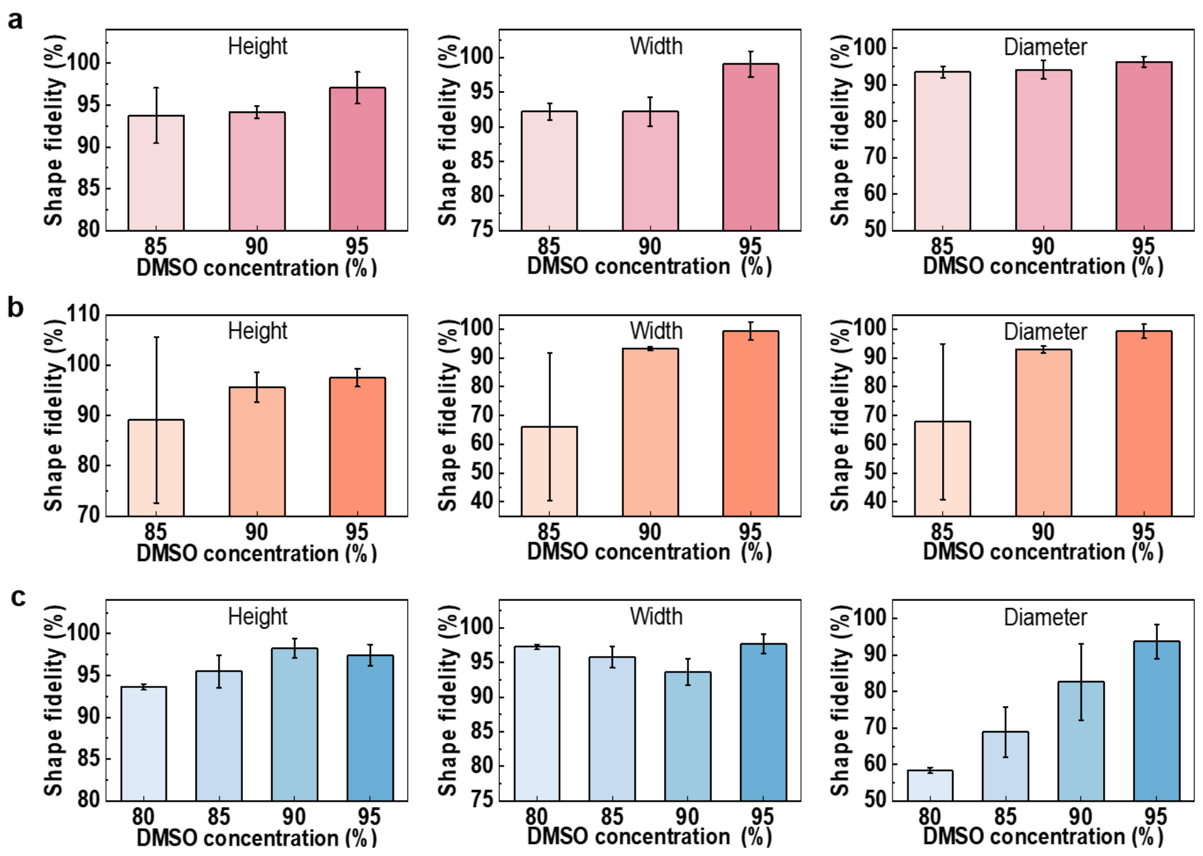

Figure S7. Effects of support material formulation (water content) on printed part quality. Shape fidelity data in terms of sample height, width, and diameter compared with design for **a,** TPU **b,** PAN, and **c,** ABS ink.

The impact of solvent concentration is particularly noticeable in ABS structures. Higher concentrations, such as 95% DMSO, effectively enhance shape fidelity by facilitating better filament fusion, as shown in the SEM images from **Figure 1f** of the main content and the shape fidelity assessments in **Figure S7c**. This improved fusion leads to a more accurate replication of the designed structures. However, at the highest concentration of 100% DMSO, there is a risk of challenging phase separation during solidification, which can distort the structure. This underscores the need to carefully balance solvent concentration to ensure accurate and structurally sound 3D-printed models.

Supplementary Note 4. Post-printing treatment and processing

Supplementary Note 4.1. Post-processing

The support-bath-enabled printing technology uses fluidic materials to create structures that must remain undisturbed in a yield-stress pre-coagulation support bath to maintain shape. Solvent loss during printing leads to material solidification but can be slow if the bath has high solvent content, resulting in soft, slowly coagulating structures. Consequently, printed structures coagulate slowly in the yield-stress pre-coagulation support bath and remain soft. To address this, a post-processing step involves immersing the bath in a non-solvent (water) reservoir, accelerating coagulation by increasing the non-solvent concentration around the object, thus facilitating solidification. To highlight the importance of post-processing, two *Y*-shaped tubular structures with 30 w/v% ABS were 3D printed. The one treated in coagulation water bath fully solidified, becoming rigid enough for successful extraction. In contrast, the structure left in the original yield-stress pre-coagulation support bath remained too soft, distorting during removal, as shown in **Figure S8**.

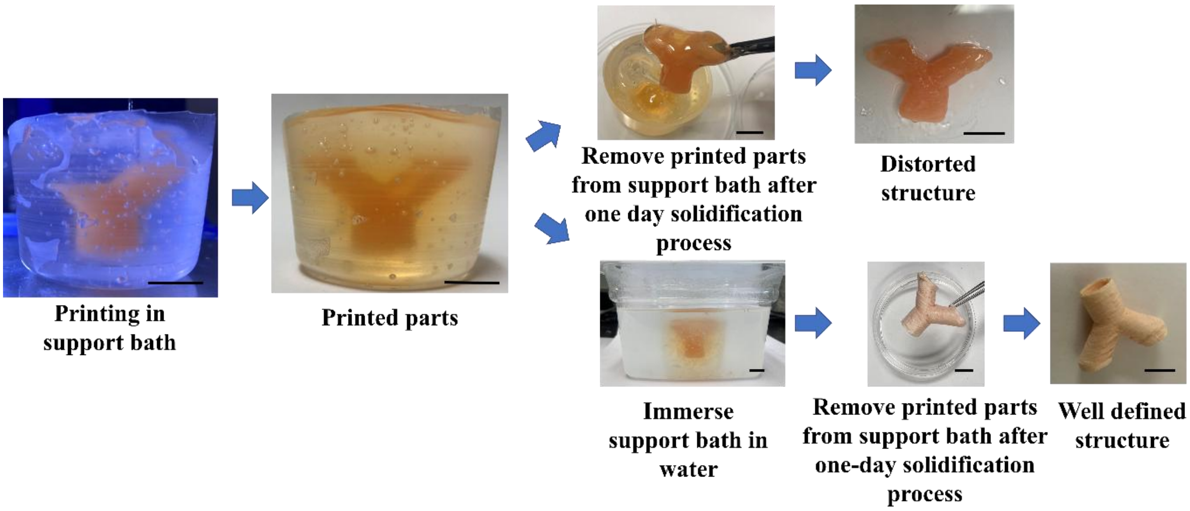

Figure S8. Comparison of post-processing approaches. Printed 30 w/v% ABS parts during and after printing in yield-stress pre-coagulation support bath material, showing contrast between parts removed before aqueous post-treatment and after aqueous post-treatment. Scale bars: 10 mm.

Supplementary Note 4.2. Dimensional fidelity of printed polymer parts

To evaluate the dimensional fidelity of the printed structures using ABS (**Figure S9a**) inks, *Y*-shaped tubular structures were printed, and their geometries were measured and compared to the original 3D designs. The surface finish of 3D-printed ABS objects can be improved using a common post-processing technique called acetone polishing. In this process, the printed ABS parts are placed in a sealed container with a small amount of acetone, which generates a vapor that uniformly melts the surface of the part. We also evaluated the dimensional accuracy of the acetone post-processed ABS parts (shown in **Figure S9b**).

Recycling plastics and polymers is crucial due to the limited fossil-based resources on Earth and the negative impact on the environment caused by our actions. As a result, sustainable plastic management has become one of the greatest challenges of our time. **Figure**

S9c shows that recycled ABS parts are redissolved and used for printing to demonstrate the concept of eco-printing and circular economy. All of the printed structures were compared to their original 3D designs in terms of various dimensions such as diameter, height, inclination angle, length, and width, and any slight discrepancies were primarily attributed to ink diffusion. Overall, no significant differences were observed between the designed models and the printed 3D structures. The dimensional fidelity results are summarized in **Figures S9d–f**.

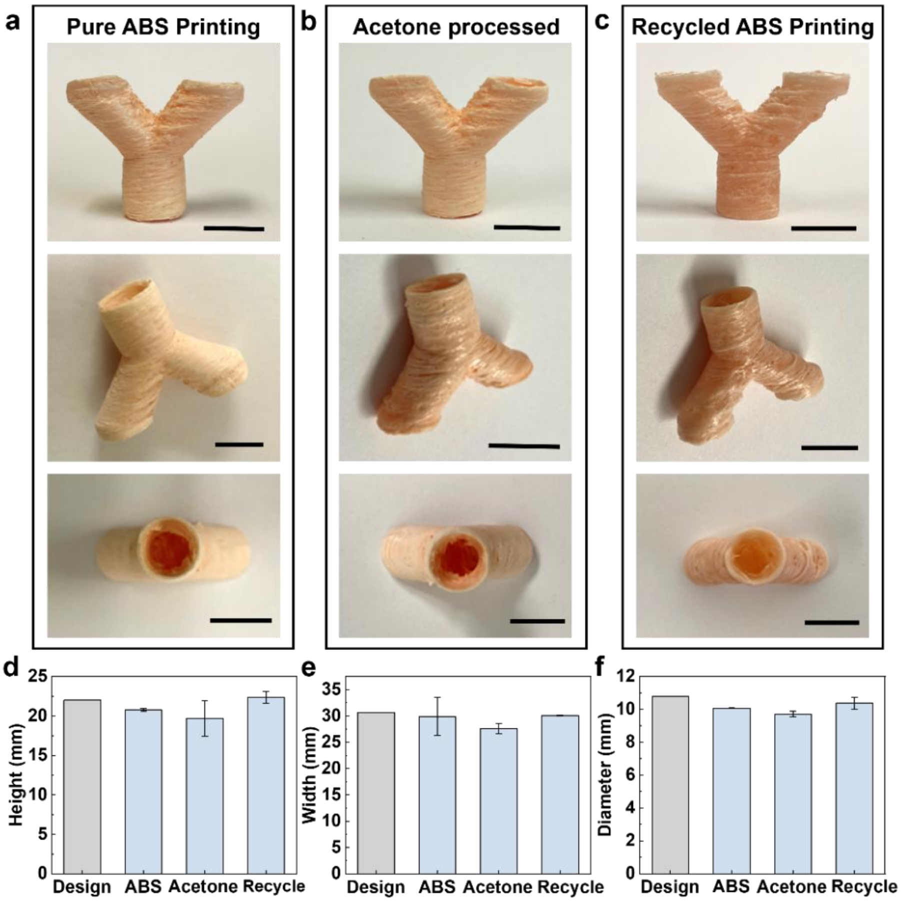

Figure S9. a, ABS printed, **b,** acetone post-processed, **c,** recycled ABS-printed *Y*-shaped tubular structures. Scale bars: 10 mm. Comparison of dimensions between designed 3D models and printed 3D structures according to **d,** height, **e,** width, and **f,** tube diameter.

Supplementary Note 5. Rheology properties of yield-stress pre-coagulation support baths

The coagulation rate of printed objects is mainly influenced by the solvent composition in the yield-stress pre-coagulation support bath, which drives solvent diffusion. Meanwhile, the concentration of the rheology modifier, such as Carbopol, critically affects the overall rheology of the bath and consequently influences print quality. Although solvent composition plays a crucial role in printing performance, it minimally impacts the yield stress and shear thinning properties of the bath. **Figure S10** demonstrates that 1.5% Carbopol 940 maintains similar rheological behavior in 85%, 90%, and 95% DMSO solutions. This is a significant advantage of the three-component system examined in this study, as the rheology can be adjusted independently of the solvent composition, which controls coagulation, and vice versa. However, higher concentration of Carbopol (2.0% and 2.5% in 95% DMSO) results in increased yield stress.

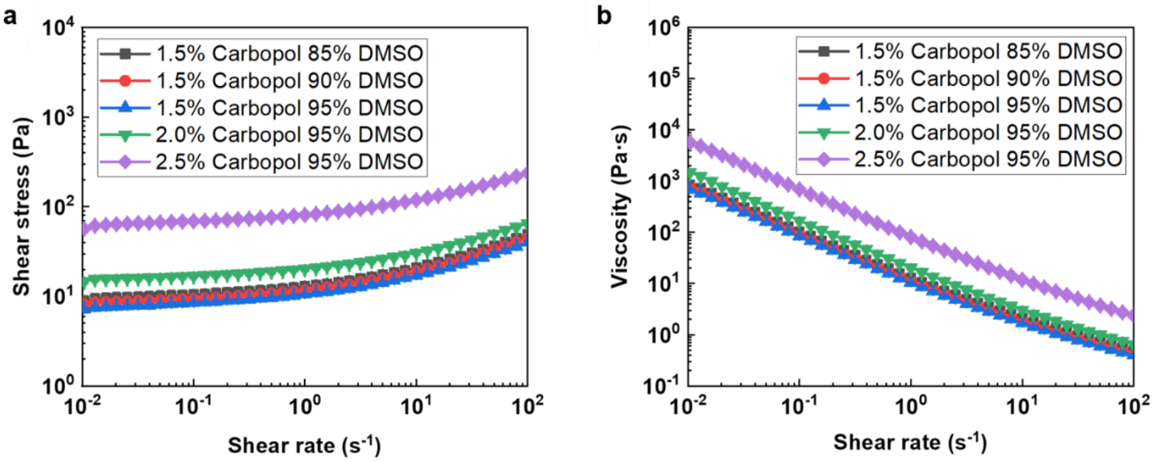

Figure S10. Support bath material rheology measurements for Carbopol-based formulations. **a**, Steady shear rate sweep data showing plateau at low shear rate. **b**, Steady shear flow curve showing shear thinning behavior.

The Herschel–Bulkley model was employed to accurately characterize and contrast the behaviors of different yield-stress support bath formulations, as depicted in **Supplementary Table 3**. This model represents a viscoplastic non-Newtonian approach, amalgamating the yield-stress impact from the Bingham model and the power-law fluids' shear-thinning or shear-thickening behavior. The relationship is given by:

$$\sigma = \sigma_0 + K\dot{\gamma}^n \quad \text{(S5-1)}$$

where σ is the total stress, σ_0 is the yield stress, $\dot{\gamma}$ is the shear rate, and K and n are the adjustment parameters. Here, n serves as the flow index, reflecting the degree of shear thinning or thickening; $n = 1$ for materials with a constant viscosity, less than 1 for shear-thinning, and greater than 1 for shear-thickening materials.

Supplementary Table 3. Herschel–Bulkley fitting parameters for various yield-stress pre-coagulation support bath formulations

Material	σ_0 (Pa)	K	n
1.5% Carbopol in 85% DMSO	8.9 ± 1.2	4.2 ± 0.4	0.47 ± 0.02
1.5% Carbopol in 90% DMSO	7.7 ± 0.4	4.5 ± 0.2	0.42 ± 0.01
1.5% Carbopol in 95% DMSO	6.7 ± 0.1	4.2 ± 0.2	0.41 ± 0.01
2.0% Carbopol in 95% DMSO	15.34 ± 0.17	4.8 ± 0.17	0.51 ± 0.01
2.5% Carbopol in 95% DMSO	61.66 ± 1.0	18.99 ± 1.0	0.48 ± 0.01

Supplementary Note 6. Ink materials and printing parameters

Four different polymeric materials and the corresponding composite materials based on these polymeric materials are utilized as build materials. For specific ink and bath formulations, printing parameters and purpose of use are summarized in **Supplementary Table 4**. When dissolved with the solvent (DMSO), all these polymeric inks exhibit shear thinning properties (ABS, TPU, and PAN) or relatively low viscosity (PCL), making them suitable for extrusion printing (as shown in **Figure S11**).

Supplementary Table 4. Ink formulations and printing parameters

Ink type	Build ink material	Ink concentration (w/v%)	Support bath		Printing parameter			Purpose
			Carbopol (w/v%)	DMSO (v/v%)	Nozzle diameter (mm)	Printing speed (mm/s)	Flow rate (mm^3/s)	
Polymer-based ink	ABS	30%	2.0	95	0.33	2	1.0	Thermoplastic copolymer for rigid part printing and comparison to conventional FDM
	TPU	50%	2.0	90	0.33	2	1.0	Soft thermoplastic polymer for flexible part printing to demonstrate versatility of approach
	PAN	15%	2.0	90	0.33	2	1.0	Work as the binder for metal printing
	PCL	27.5%	1.5	90	0.25	2	0.35	Biocompatible material widely used in tissue engineering
Polymer-based composite ink	ABS/ TPU	36%/ 11%	1.5, 2.0	90	0.25	4, 5	0.5, 1.2	Adjustable strength and flexibility
	ABS/ carbon fiber	30%/10%	2.0	95	0.91	2	1.0	For mechanical property enhancement
	PAN/ stainless steel	15%/75%	3.0	90	0.91	2	1.0	Metallic biomaterial widely used in surgical implantation and other biomedical applications

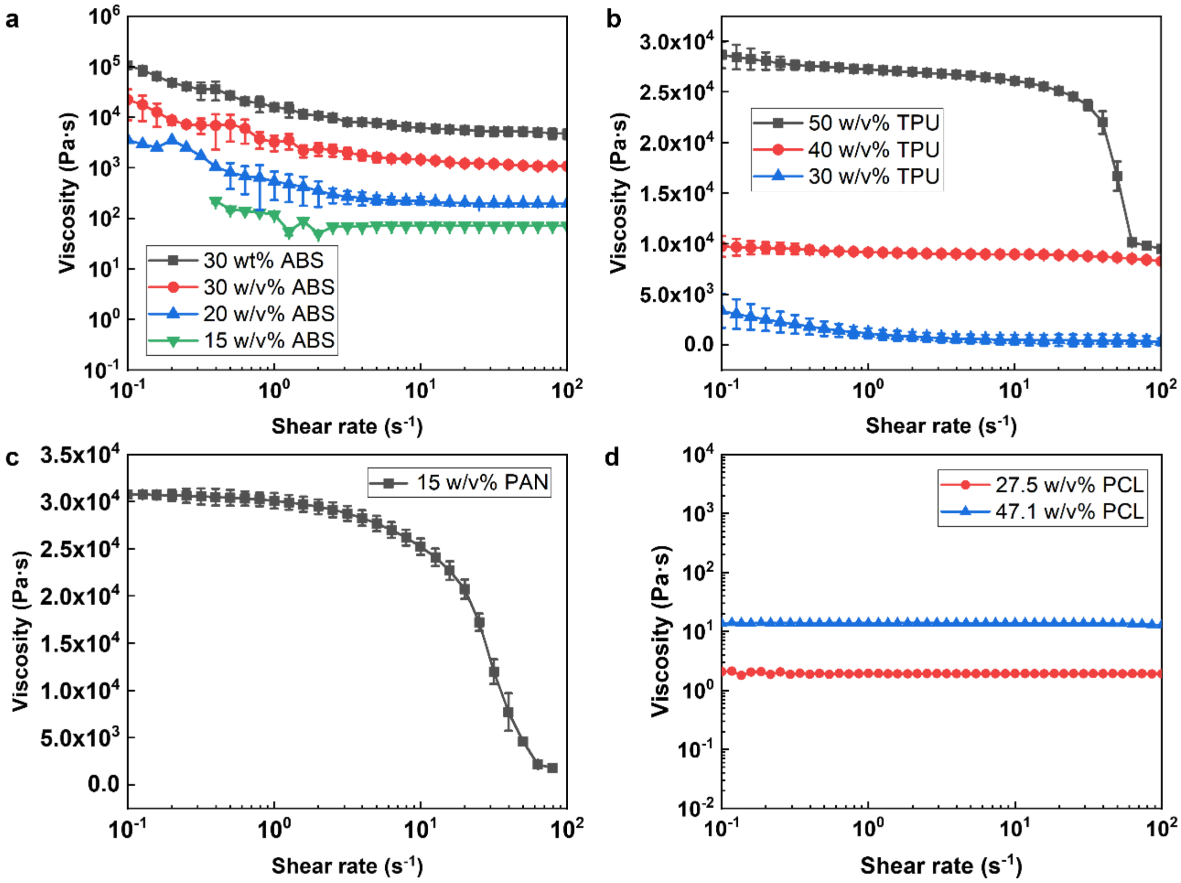

Figure S11. Apparent viscosity under steady shear of different polymer inks. a, ABS, **b**, TPU, **c**, PAN, **d**, PCL ink.

Supplementary Note 7. Additional printed parts

Supplementary Note 7.1. Additional printed PCL parts

Additional structures fabricated using PCL, including single filament-based truss structures and solid parts, are depicted in **Figure S12**. The lattice structures, shown in **Figures S12a** and **S12b**, demonstrated effective fusion at the junctions between adjacent points, as visibly confirmed by the SEM image of the peak. This resulted in 3D structures with negative Poisson's ratios and anti-four-chiral configurations—complex geometries that are challenging to achieve with traditional extrusion printing methods in the air. These structures provided considerable flexibility, enabling them to deform easily and return reliably to their original shape under various stress conditions such as tension, expansion, and compression.

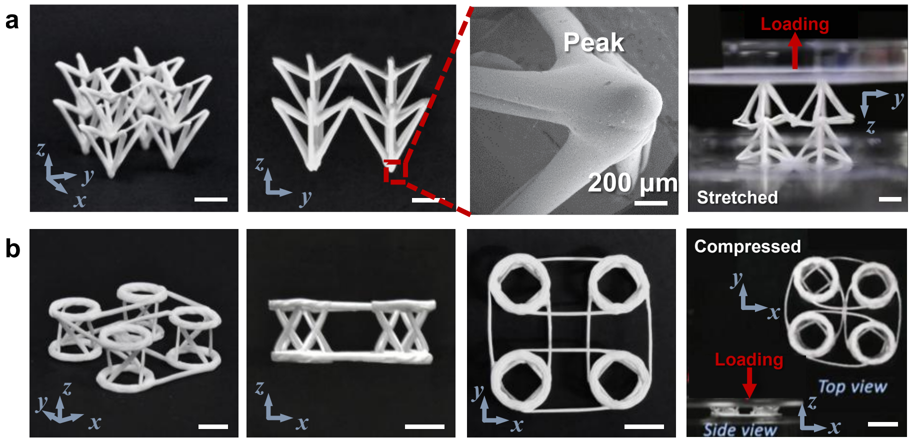

Figure S12. Printed PCL parts. a, 3D structure with negative Poisson's ratio. **b,** 3D anti-four-chiral configuration. Scale bars: 5 mm.

Supplementary Note 7.2. ABS/TPU composite structures printing

The use of a yield-stress pre-coagulation support bath in this process eliminates the need for traditional support structures, thereby reducing material waste and avoiding surface quality degradation after support removal. **Figure S13** showcases the effectiveness of this method in freeform printing. For instance, the lasso structure in **Figure S13a** features a regular hexahedron as an independent unit, while **Figure S13b** highlights a complex three-layer ghost ball known for its intricate design and precision. Such detailed structures, particularly those where internal components like the sleeve ball cannot be removed, are challenging to achieve with traditional extrusion due to the necessity of supports. This innovative printing process simplifies the production of complex structures by utilizing the support capabilities of the yield-stress pre-coagulation support bath and its easy cleanup. The method extends to a variety of challenging structural models that are difficult to achieve with conventional polymer extrusion. For example, **Figures S13c–d** display spiral plates and C20 fullerenes structures.

Figure S13e demonstrates the first-order Hilbert channel, characterized by smooth internal and external surfaces and excellent interlayer bonding. The channel effectively allows deionized water to flow from one end to the other without leakage, showcasing the potential of this method for creating precise and functional fluid channels.

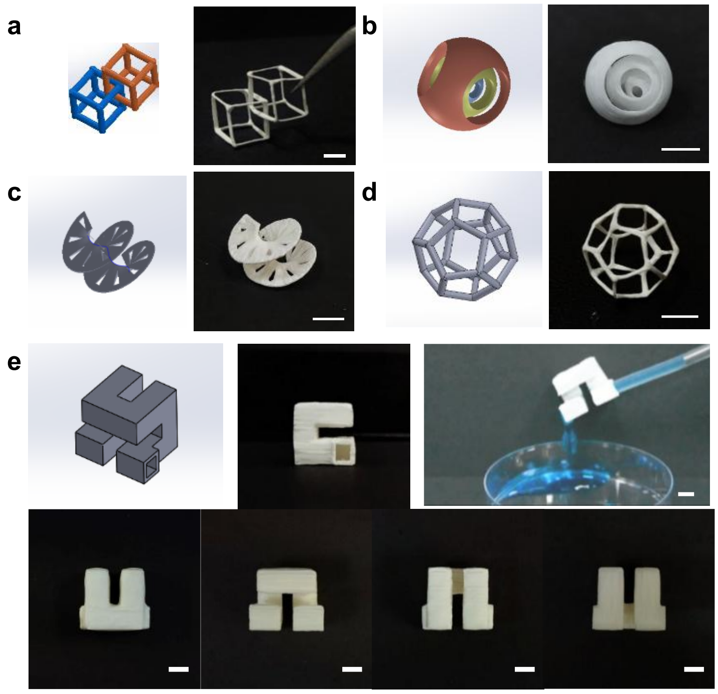

Figure S13. Printed model and pictures of ABS/TPU **structures. a,** regular hexagonal lasso structure; **b,** ghost ball, **c,** spiral plate, **d,** C20 fullerene, **e**, first-order Hilbert channel structure. Scale bars: 5 mm.

Negative Poisson’s ratio, often referred to as auxetic behavior, describes a material that expands laterally when stretched and contracts laterally when compressed within its elastic range. This unique characteristic leads to various structural categories based on the deformation mechanism, including concave polygon, rotating rigid body, chiral, perforated plate, nodal fiber, interlocking polygon, and fold structures. **Figure S14** illustrates examples of negative Poisson's ratio structures produced using this process. Leveraging the elastic properties of polyurethane, the structure achieves auxetic behavior both in two-dimensional and three-dimensional forms. The figure also highlights the deformation trends of specific structures such as the positive chiral, concave hexagon, and fold, demonstrating the versatile potential of this innovative printing technique in realizing complex auxetic geometries.

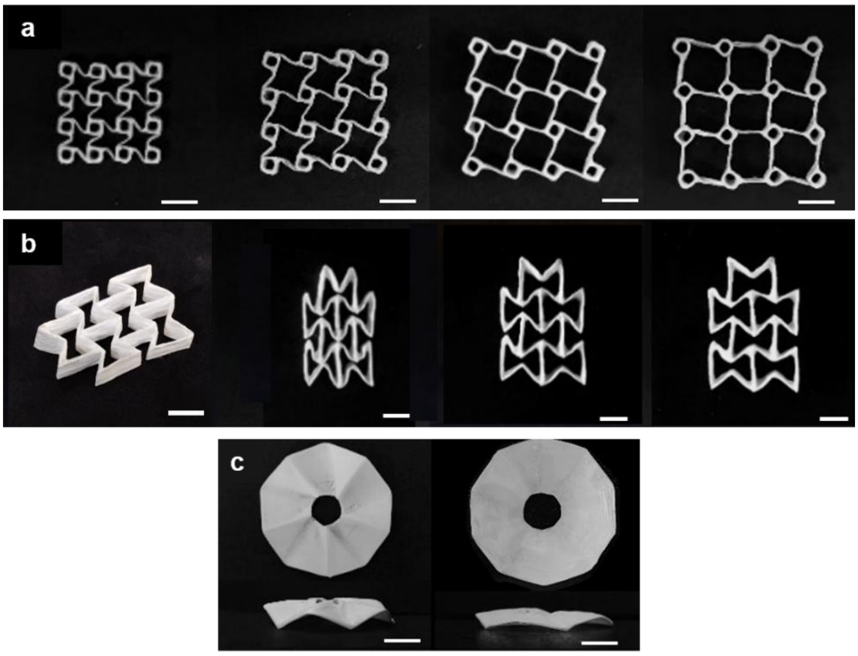

Figure S14. Structures with negative Poisson's ratio fabricated by IPS-E3DP. Deformation tendency of a, Orthochirality, **b,** concave hexagon and **c,** fold structure. Scale bars: 5 mm.

Supplementary Note 8. Effects of ink formulation on pore size and mechanical properties

In polymer-based ink printing, the interaction between solvent and non-solvent shapes micro-porous structures, influenced by polymer concentration and solvent ratios in the yield-stress pre-coagulation support bath. This process allows for the fabrication of polymer structures with specific microscopic pores and macroscopic mechanical properties, enhancing features like biocompatibility, biodegradability, and strength. To assess the influence of polymer concentration on these properties, we conducted relevant experimental studies, and the results are as follows:

Supplementary Note 8.1. PCL printing results

The SEM results of printed PCL, shown in **Figure S15a**, demonstrate distinct differences between samples produced using our IPS-embedded 3D printing (IPS-E3DP) method and traditional FDM.

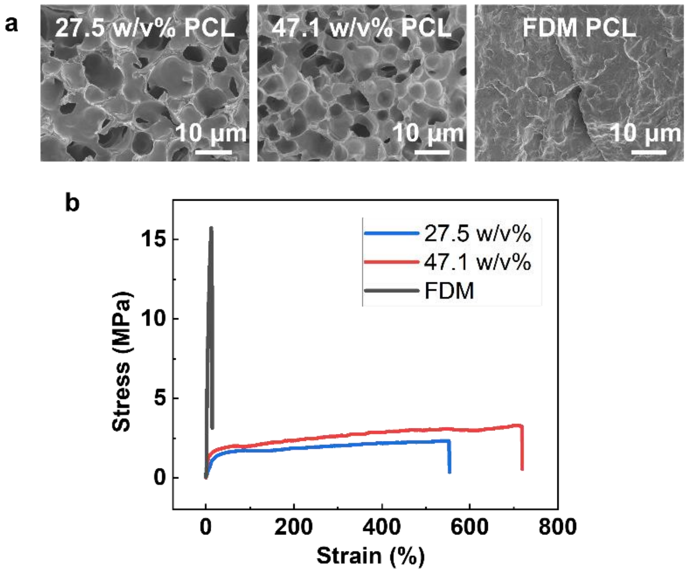

Figure S15. Pore size and mechanical properties affected by PCL ink formulation. a, SEM images, **b**, tensile test results.

The IPS-E3DP method consistently produced uniform microscopic pores, whereas FDM did not. At lower PCL concentrations (27.5 w/v%), larger and more irregular pores formed due to faster phase separation. At higher concentrations (47.1 w/v%), pores were smaller and more uniform due to slower dynamics and stronger molecular interactions. These structural differences significantly affected mechanical properties. Filaments produced by IPS-E3DP showed enhanced tensile toughness, with fracture strains exceeding 500%, greatly surpassing the 13% observed in FDM-printed filaments (**Figure S15b**). This improvement is attributed to the microscopic pore structure which effectively absorbs and disperses stress, thereby lowering Young's modulus and maximum tensile stress compared to FDM-printed PCL. These findings highlight the critical role of pore control in optimizing the mechanical performance of 3D printed materials.

Supplementary Note 8.2. ABS printing results

For ABS inks at 15%, 20%, and 30% concentrations, all can produce printable parts, but the structures made from 15% and 20% concentrations are less robust, as shown in **Figure S16a**. The reduced stiffness at these lower concentrations is due to the formation of larger pores, a result of phase separation when fewer polymer molecules are available to form a dense network. This leads to weaker intermolecular forces and a diminished capacity to resist coagulation when exposed to non-solvent, resulting in looser structures with larger void spaces. As shown in **Figures S16a** and **b**, these larger pores occupy a relatively large volume fraction of the material, resulting in higher porosity. The presence of voids reduces material density, weakens the structural integrity, and decreases load-bearing capacity, ultimately leading to lower mechanical properties (as seen in **Figure S16c**).

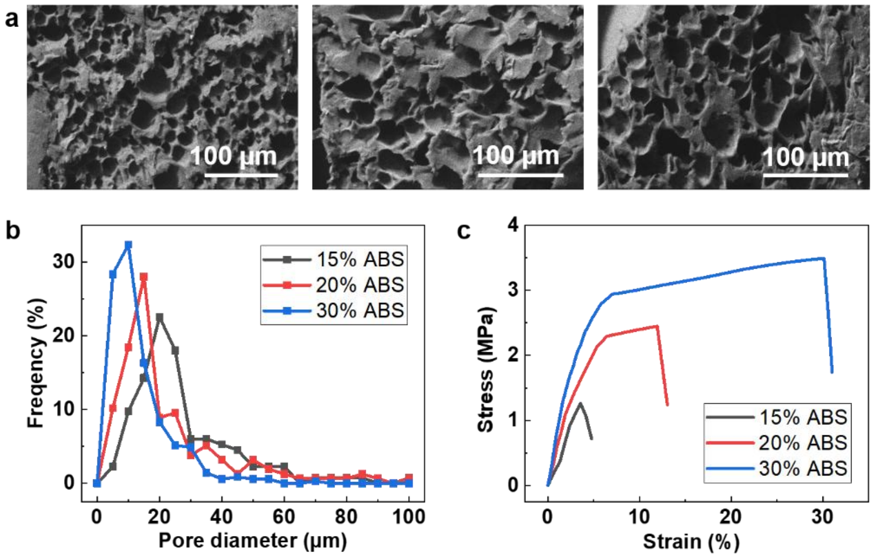

Figure S16. Pore size and mechanical properties affected by ABS ink formulation. a, SEM images, **b,** pore size distribution, and **c,** tensile test results of samples printed with ABS ink at different concentrations.

Supplementary Note 8.3. TPU printing results

TPU inks, used in concentrations from 20% to 50%, can also produce identifiable parts that are capable of supporting their own weight in air (**Figure 5c**), they exhibit different pore sizes (as depicted in **Figure S17a**) which leads to distinct mechanical properties (as shown in **Figure S17b**). Thus, the relationship between pore size and mechanical properties through ink concentration highlights our ability to regulate micropores and mechanical properties of pure polymer parts.

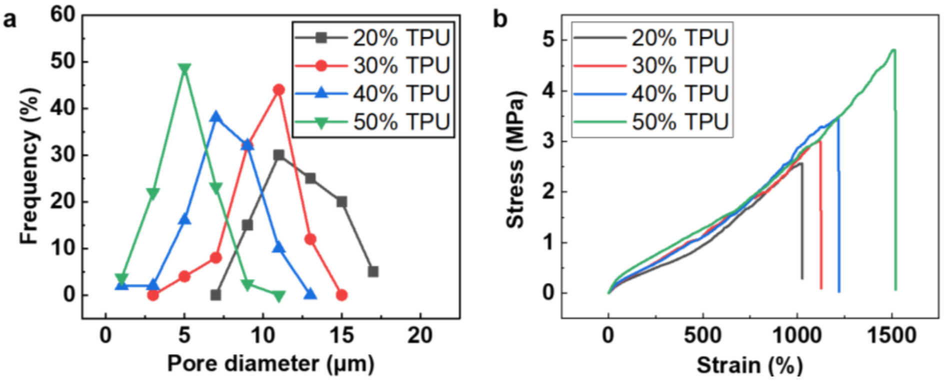

Figure S17. a, Pore size distribution and **b,** Tensile test results of TPU parts.

Supplementary Note 8.4. ABS/TPU printing results

To find the optimal soft polymer ink for auxetic structures, we evaluated the print quality and mechanical properties of various ABS/TPU ink formulations. **Figure S18a** shows that 47 w/v% ABS ink filaments were oversized, while 19 w/v% TPU ink produced rough filaments with visible pores. In contrast, ABS/TPU blends yielded smoother, uniformly-sized filaments.

Uniaxial tensile tests detailed in **Figure S18b** and **Supplementary Table 5** revealed that 47 w/v% ABS filaments had the highest Young's modulus (346.97 ± 36.91 MPa) and fracture strength (11.44 ± 1.91 MPa), but the lowest fracture strain of only about 5%. Meanwhile, 19 w/v% TPU filaments exhibited a high fracture strain of approximately 850%, but insufficient modulus and strength for standalone structures. The 36 w/v% ABS/11 w/v% TPU blend combined excellent printability with robust mechanical properties (Young's modulus ~280 MPa, fracture strain ~130%), making it the optimal choice for printing durable auxetic structures.

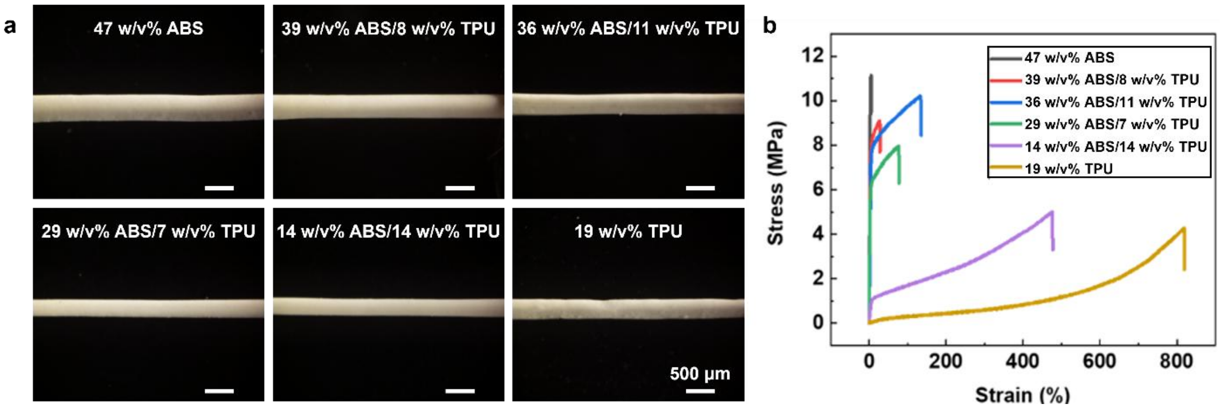

Figure S18. Printability and mechanical properties affected by ink formulation. a, Single filaments printed with ABS/TPU composites. **b,** Tensile test results of ABS/TPU filaments, respectively.

Supplementary Table 5. Mechanical properties of printed ABS/TPU filaments from tensile testing

Ink formulation (w/v%)	Young's modulus (MPa)	Fracture strength (MPa)	Fracture strain (%)
47% ABS	346.97 ± 36.91	11.44 ± 1.91	4.90 ± 0.82
39% ABS/8% TPU	280.77 ± 26.43	8.81 ± 0.61	21.58 ± 3.52
36% ABS/11% TPU	279.64 ± 2.59	10.56 ± 0.35	130.18 ± 2.08
29% ABS/7% TPU	199.75 ± 6.15	7.55± 1.17	72.10 ± 10.95
14% ABS/14% TPU	26.10 ± 2.51	5.04 ± 0.07	470.86 ± 27.89
19% TPU	1.32 ± 0.14	4.24 ± 0.17	862.62 ± 205.81

Supplementary Note 9. Mechanical property for carbon fiber reinforced ABS parts

Figures S19a and **b** demonstrate an increase in Young's modulus and tensile strength with increasing carbon fiber concentration (from 0 w/v% to 15 w/v%).

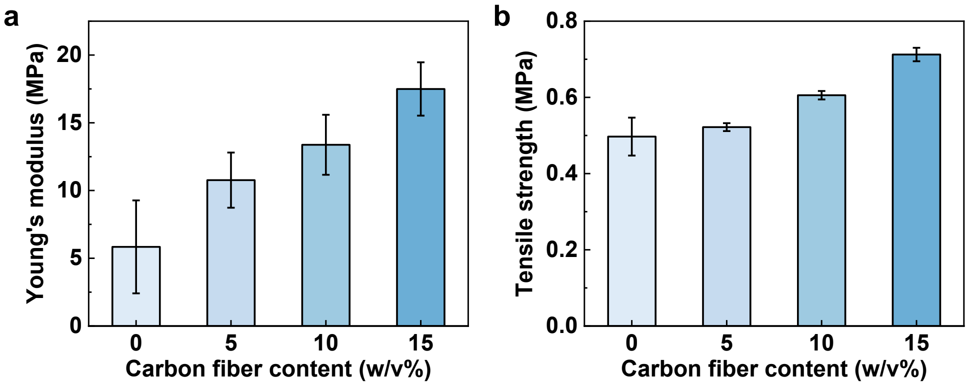

Figure S19. a, Young's modulus and **b,** tensile strength evaluations.

Supplementary Note 10. Polymer-assisted metal printing

Supplementary Note 10.1. Debinding and sintering conditions

To achieve densification of the printed stainless-steel parts and remove the polymer binder, a sintering process was carried out in a furnace oven. The sintering procedure involved heating and cooling rates of 5 °C /min and a dwell time of 30 minutes at 300 °C to prevent thermal cracking on the alumina tube. The debinding cycle was determined based on the Thermogravimetric analysis (TGA) data and set at 500 °C for 60 minutes as the binder polymer PAN undergoes rapid mass loss in the 270–300 °C range and moderate mass loss in the 300–470 °C range. The temperature profile of the furnace is illustrated in **Figure S20**.

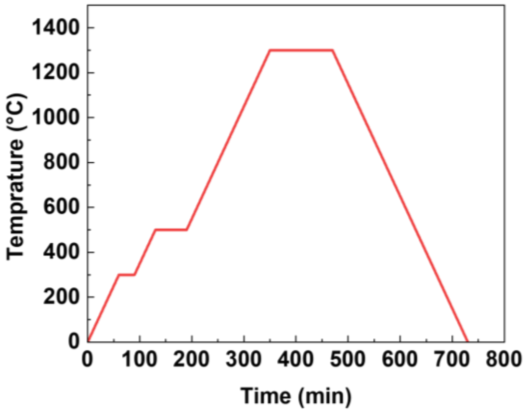

Figure S20. Thermal profile for the debinding and sintering of the PAN-316L-based printed parts.

Supplementary Note 10.2. Dimensional fidelity of printed metal parts

To evaluate the print accuracy of the metal structures, *X*-shaped, *Y*-shaped, and *L*-shaped overhang metal parts were 3D printed and compared with the designed 3D models after sintering. The dimensions of the designed models and the printed 3D structures, including height (**Figure S21a**), width (**Figure S21b**), and tube diameter (**Figure S21c**), were compared and found to be consistent with the designed models. However, after sintering and sandblasting, the shrinkage of the structures was approximately 80% due to the removal of the binder material (15 w/v% PAN).

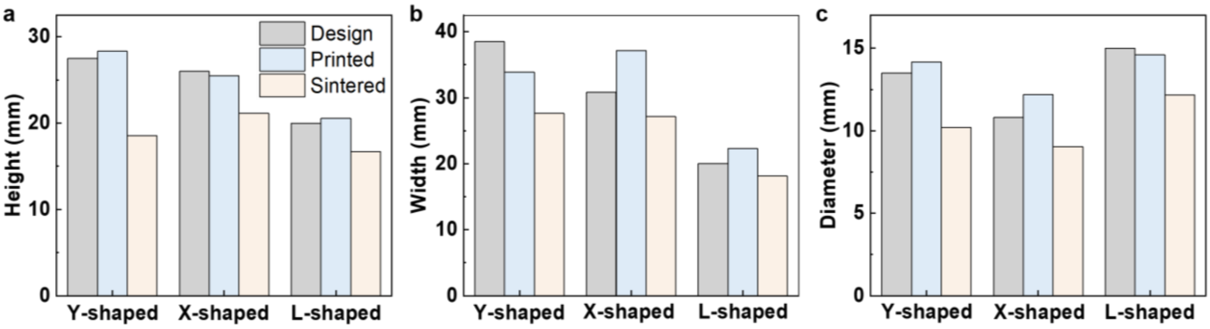

Figure S21. Comparison of dimensions among designed 3D models, printed 3D structures, and sintered structures in terms of a, height, b, weight, and c, diameter.

Supplementary Note 10.3. Metal part evaluation

To assess the composition of stainless-steel parts before and after sintering, the parts were sectioned, embedded in resin, polished, and examined using SEM. The comparison between

Figure S22a (structure with voids) and **Figure 5a** (structure without voids) demonstrates the densification of printed parts during the sintering process. Additionally, by comparing the EDS results of the parts before (**Supplementary Table 6** and **Figure S22b**) and after the sintering process (**Supplementary Table 7** and **Figure S22c**), it was discovered that there was more carbon present before sintering, indicating that PAN was removed during the sintering process.

Supplementary Table 6. Unsintered sample

	Element Number	Element Symbol	Element Name	Atomic Conc.	Weight Conc.	Oxide Symbol	Stoich. Weight Conc.
	6	C	Carbon	19.356	5.205		
	8	O	Oxygen	6.706	2.402		
	14	Si	Silicon	1.591	1.001		
	24	Cr	Chromium	11.004	12.813		
	26	Fe	Iron	50.348	62.963	Fe_3O_4	100.000
	28	Ni	Nickel	9.597	12.613		
	42	Mo	Molybdenum	1.398	3.003		

Supplementary Table 7. Sintered sample

	Element Number	Element Symbol	Element Name	Atomic Conc.	Weight Conc.	Oxide Symbol	Stoich. Weight Conc.
	6	C	Carbon	7.340	1.698		
	8	O	Oxygen	2.917	0.899		
	24	Cr	Chromium	12.864	12.887		
	26	Fe	Iron	63.969	68.831	Fe_3O_4	100.000
	28	Ni	Nickel	11.397	12.887		
	42	Mo	Molybdenum	1.513	2.797		

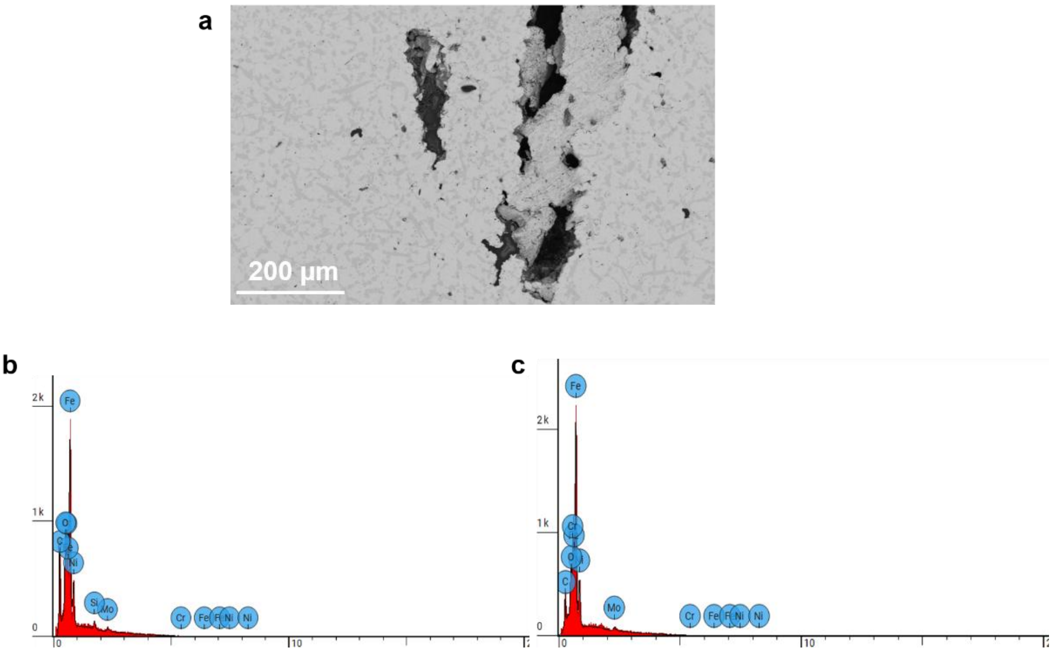

Figure S22. a, SEM image of printed part's cross-section without sintering. EDX readings of 316L printed part's cross-section **b,** without and **c,** with sintering.

Supplementary Note 11. TPU-based adsorber analysis

The SEM image of biochar in **Figure S23a** indicates that it provides a higher surface area, which can enhance the adsorber's performance. Furthermore, **Figure S23b** depicts the mineral oil uptake rate for solid and lattice TPU adsorbers. The lattice adsorbers' larger surface area significantly reduces diffusion time and enhances oil transfer efficiency, resulting in relatively rapid kinetic uptake (approximately 5 seconds, compared to 30 seconds for solid adsorbents).

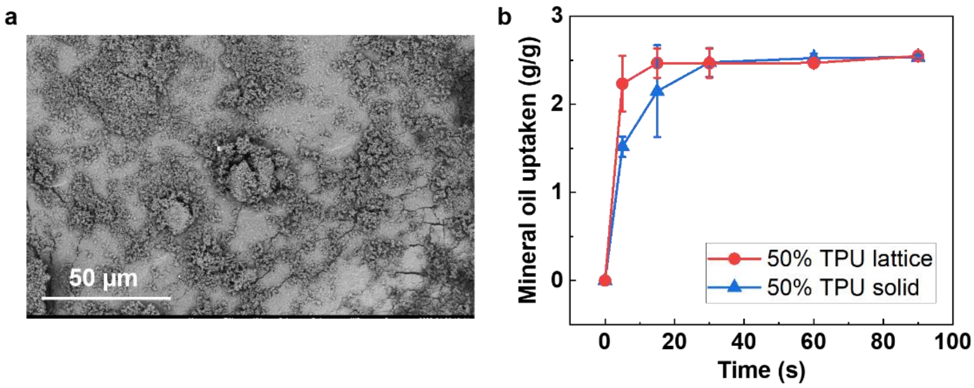

Figure S23. a, SEM image of biochar. c, Mineral oil uptaken speed comparison.

Supplementary Note 12. Porous structure biomineralization

Supplementary Note 12.1. Microbially-induced mineral growth mechanism

Microbially-induced mineral growth (MIMG) involves the formation of minerals through the activities of microorganisms like bacteria, fungi, and algae. As illustrated in **Figure S24**, *Sporosarcina pasteurii* (*S. pasteurii*) uses urease to convert urea into ammonia and carbon dioxide, increasing pH and providing carbonate ions for calcium carbonate ($CaCO_3$) precipitation. Calcium ions adhere to the bacterium's negatively charged surface, initiating crystal formation.[9,10]

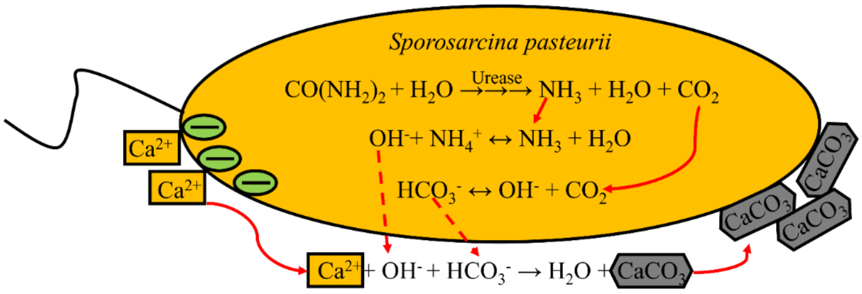

Figure S24. Schematic of microbially-induced mineral growth (MIMG) catalyzed by *S. pasteurii*.

To evaluate biomineralization speed, 3 × 3 lattice structures were produced using FDM and compared to those made with the IPS-E3DP process. Results (**Figure 6c**) indicated that IPS-E3DP parts had a significantly higher biomineralization rate due to their multi-scale porous structures, enhancing calcium ion adsorption and calcium carbonate crystal growth. To compare the mineral volume fraction with 3 × 3 lattice structures ($BR = 0.5$ mm and $b = 2$ mm) (**Figure 6c**), **Figure S25a** displays IPS-E3DP printed 2 × 2 lattice structures ($BR = 0.5$ mm and $b = 3$ mm). The normalized mineral volume fraction for a cubic unit cell with a beam diameter of $2BR$, unit size of b, and mineral thickness of H can be calculated using Equation (S12-3). As illustrated in **Figure 6d**, the results indicate that the mineral volume fraction increases more rapidly as the unit cell size decreases. This can be attributed to the fact that a smaller unit cell size corresponds to a larger relative internal surface area, which promotes the MIMG process. SEM images (**Figure S25b**) reveal that the biomineralized parts possess a highly porous structure that is composed of printing routes-defined macropores, inter-filament mesopores, and inherent micropores within a multi-scale porous structure.

To model mineral growth for prediction purposes, we used an established model.[11] This model describes the relationship between the normalized mineral thickness and time using the following equation:

$$\frac{d(H/BR)}{d(Dt/BR^2)} = \frac{1}{\frac{c_p}{c_m}\left(\sqrt{\left(\frac{H}{r}+1\right)^2\left(\frac{2c_p}{c_m}-1\right)-\left(\frac{2c_p}{c_m}-2\right)}-H/BR-1\right)} \quad \text{(S12-1)}$$

here, c_p is the solid mineral molar concentration of calcium carbonate, c_m is the molar concentration of calcium carbonate in the oversaturated solution, D is the diffusion coefficient of calcium carbonate within the solution, r is the distance from the center of the lattice beam. The normalized mineral thickness can be written as a function of time t as:

$$H/BR = G\left(Dt/BR^2, \frac{c_p}{c_m}\right) \quad \text{(S12-2)}$$

Using the experimental parameters and estimated parameters $\frac{c_p}{c_m} = 1.7$, $D = 4.8 \times 10^{-8}$, we can obtain the relationship between *H/R* and mineralization time. The mineral volume fraction is calculated as:

$$\varphi\,(H) = \frac{\left[8\left(1+\frac{H}{BR}\right)^3 + 12\left(\frac{b}{BR} - 2 - \frac{2H}{BR}\right)\left(1+\frac{H}{BR}\right)^2\right] - \left[\frac{12b}{BR} - 16\right]}{\left(\frac{b}{BR}\right)^3} \quad \text{(S12-3)}$$

When the void is fully covered, the maximum mineral thickness is:

$$H_{max} = \frac{b}{2} - BR \quad \text{(S12-4)}$$

The normalized volume fraction can be calculated as:

$$\eta = \frac{\varphi(H)}{\varphi(H_{max})} \times 100\% \quad \text{(S12-5)}$$

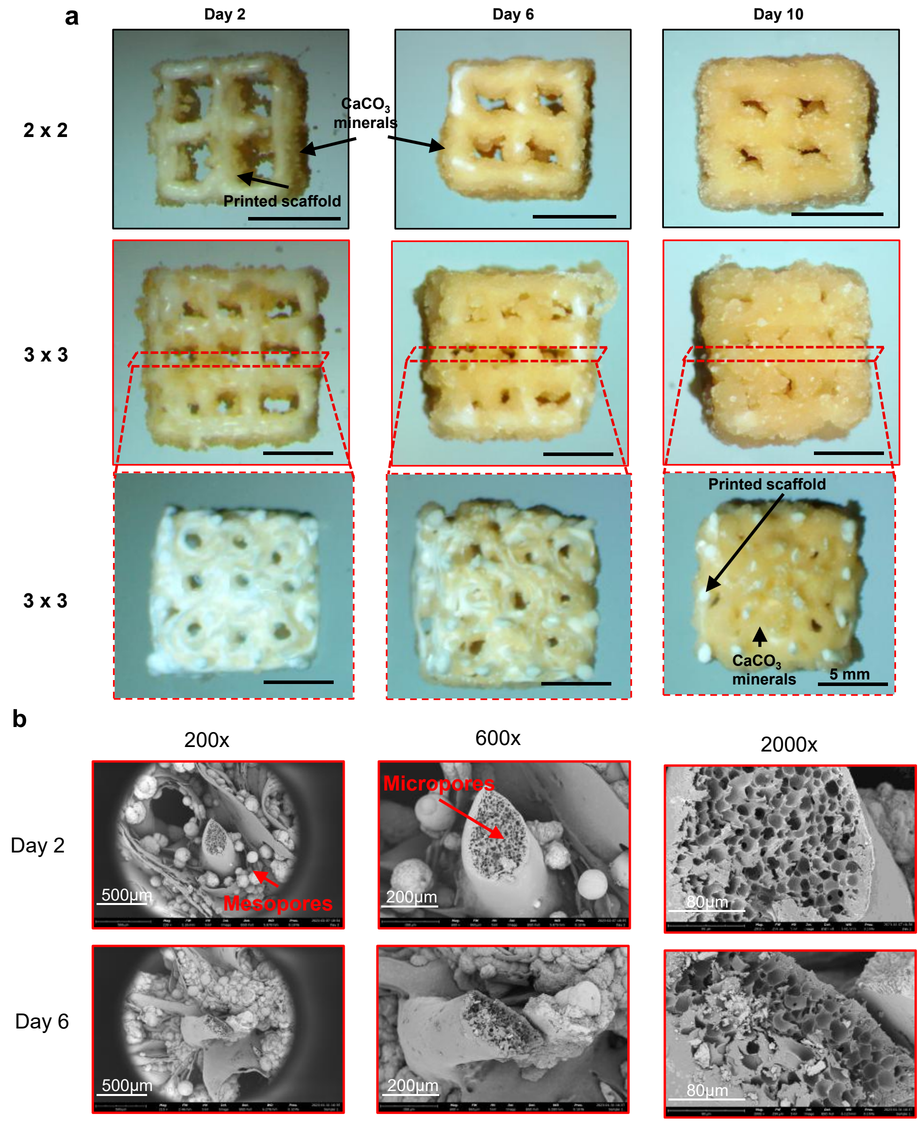

Figure S25. Comparison of biomineralization process. a, 2 × 2 and 3 × 3 cube lattice parts under biomineralization. **b,** SEM images of cross-section of biomineralized 3 × 3 cube lattice from Day 2 to Day 6.

Supplementary Note 12.2. Compression test of printed parts during biomineralization process

To assess the mechanical behavior of the mineralized samples during the experimental period, we conducted compressive tests. **Figure 6d** in the main content illustrates the Young's Modulus for the biomineralized parts (3 × 3 lattice structure) on different days, and the Young's modulus of each sample was calculated based on the curves. Our results demonstrate that the effective stiffness of the virgin lattice structure on day 0 was 58.09 MPa. However, after 10 days of mineralization, the effective stiffness of the sample increased to 627.05 MPa, representing a significant 10.79-fold increase (**Figure S26**).

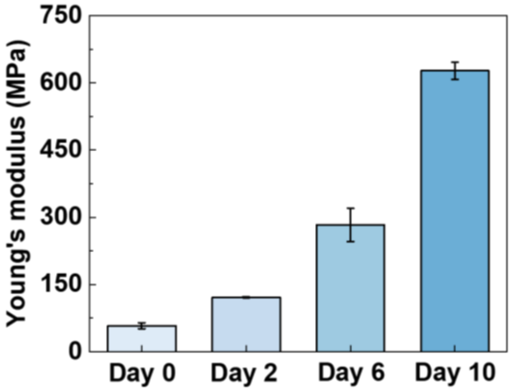

Figure S26. Compression Young's modulus of the mineralized sample over 10 days.

Supplementary Note 13. Comparison of IPS-E3DP with Conventional Additive Manufacturing Methods

Supplementary Table 8. Comparison of IPS-E3DP with conventional methods

Method	Operating Temp.	Material Compatibility	Support Requirement	Printing resolution	Build Time	Energy Consumption	Estimated Cost
IPS-E3DP	Room temperature	Engineering polymers, blends, composites	No need	50 µm	Moderate	Low	Low–Moderate
FFF[12]	High (⩾180 °C)	Thermoplastics (e.g., PLA, ABS, PEEK)	Supports needed	50–200 µm[13]	Moderate	High (heating + extrusion)	Moderate
DIW[14]	Room temperature	Pastes, hydrogels, conductive/ceramic inks	Supports needed	100 µm[15]	Moderate	Low	Low
SLA/ DLP[16,17]	Room temp + UV	Photocurable polymers	Supports needed	10 µm[13]	Fast (per layer)	Moderate (UV curing)	High

References

[1] B. Zhou, A. C. Powell, *J Memb Sci* **2006**, *268*, 150.

[2] F. Boyer, S. Minjeaud, *ESAIM: Mathematical Modelling and Numerical Analysis* **2011**, *45*, 697.

[3] L. Yilmaz, A. J. McHugh, *J Appl Polym Sci* **1986**, *31*, 2847.

[4] S. Mohsenpour, F. Esmaeilzadeh, A. Safekordi, M. Tavakolmoghadam, F. Rekabdar, M. Hemmati, *J Mol Liq* **2016**, *224*, 776.

[5] M. Charles, *Hansen Solubility Parameters*, CRC Press, **2000**.

[6] P. Peng, B. Shi, L. Jia, B. Li, *Journal of Macromolecular Science, Part B: Physics* **2010**, *49*, 864.

[7] E. Ema, "Surface Free Energy Components by Polar / Dispersion and Acid — Base Analyses ; and Hansen Solubility Parameters for Various Polymers Surface Free Energy Components by Polar / Dispersion and Acid — Base Analyses ; and Hansen Solubility Parameters for Vari," **2015**.

[8] M. J. Castro-Alonso, L. E. Montañez-Hernandez, M. A. Sanchez-Muñoz, M. R. Macias Franco, R. Narayanasamy, N. Balagurusamy, *Front Mater* **2019**, *6*, 1.

[9] Y. Wu, H. Li, Y. Li, *Microorganisms* **2021**, *9*, DOI 10.3390/microorganisms9112396.

[10] V. Guarino, A. Guaccio, D. Guarnieri, P. A. Netti, L. Ambrosio, *J Biomater Appl* **2012**, *27*, 241.

[11] A. Xin, Y. Su, S. Feng, M. Yan, K. Yu, Z. Feng, K. Hoon Lee, L. Sun, Q. Wang, *Advanced Materials* **2021**, *33*, 1.

[12] S. Maurya, B. Malik, P. Sharma, A. Singh, R. Chalisgaonkar, in *Mater Today Proc*, Elsevier Ltd, **2022**, pp. 1217–1222.

[13] X. Wang, M. Jiang, Z. Zhou, J. Gou, D. Hui, *Compos B Eng* **2017**, *110*, 442.

[14] M. A. S. R. Saadi, A. Maguire, N. T. Pottackal, M. S. H. Thakur, M. M. Ikram, A. J. Hart, P. M. Ajayan, M. M. Rahman, *Advanced Materials* **2022**, *34*, DOI 10.1002/adma.202108855.

[15] M. A. S. R. Saadi, A. Maguire, N. T. Pottackal, M. S. H. Thakur, M. M. Ikram, A. J. Hart, P. M. Ajayan, M. M. Rahman, *Advanced Materials* **2022**, *34*, DOI 10.1002/adma.202108855.

[16] E. M. Maines, M. K. Porwal, C. J. Ellison, T. M. Reineke, *Green Chemistry* **2021**, *23*, 6863.

[17] A. Amini, R. M. Guijt, T. Themelis, J. De Vos, S. Eeltink, *J Chromatogr A* **2023**, *1692*, DOI 10.1016/j.chroma.2023.463842.
